# Supplementary material for: High-fat diet-induced upregulation of exosomal phosphatidylcholine contributes to insulin resistance
Source: Nat Commun. 2021 Jan 11;12:213. doi: 10.1038/s41467-020-20500-w (PMC7801461; doi:10.1038/s41467-020-20500-w)
Supplement: Supplementary file 1 — Supplementary Information [file 41467_2020_20500_MOESM1_ESM.pdf]

Supplemental Information

High-fat diet-induced upregulation of exosomal phosphatidylcholine contributes to insulin resistance

Anil Kumar[1], Kumaran Sundaram[1], Jingyao Mu[1], Gerald W Dryden[1,2], Mukesh K Sriwastva[1], Chao Lei[1], Lifeng Zhang[1], Xiaolan Qiu[1], Fangyi Xu[1], JunYan[1], Xiang Zhang[3], Juw Won Park[4,5], Michael L Merchant[6], Henry C. L. Bohler[7], Baomei Wang[8], Shuangqin Zhang[9], Chao Qin[10], Ziying Xu[10], Xianlin Han[10], Craig J McClain[2], Yun Teng[1*], and Huang-Ge Zhang[1,11,12*]

[1] James Graham Brown Cancer Center, Department of Microbiology & Immunology, University of Louisville, KY 40202, USA

[2] Department of Medicine, University of Louisville, Louisville, KY 40202, USA

[3] Department of Pharmacology and Toxicology, University of Louisville, Louisville, KY 40202, USA

[4] Department of Computer Engineering and Computer Science, University of Louisville, KY 40202, USA

[5] KBRIN Bioinformatics Core, University of Louisville, Louisville, KY 40202, USA

[6] Kidney Disease Program and Clinical Proteomics Center, University of Louisville, Louisville, KY, USA

[7] Department of Reproductive Endocrinology and Infertility, University of Louisville, Louisville, KY40202, USA

[8] Department of Dermatology, University of Pennsylvania, Philadelphia, 19104, USA

[9] Peeples Cancer Institute, 215 Memorial Drive, Dalton, GA 30720 USA

[10] Barshop Institute for Longevity and Aging Studies, University of Texas Health Science Center at San Antonio, San Antonio, TX, 78229, USA

[11] Robley Rex Veterans Affairs Medical Center, Louisville, KY 40206, USA

[12] Lead contact

* These authors jointly supervised this work

CONTACT FOR REAGENT AND RESOURCE SHARING

Further information and request for reagents may be directed to lead contact,

Dr. Huang-Ge Zhang (h0zhan17@louisville.edu).

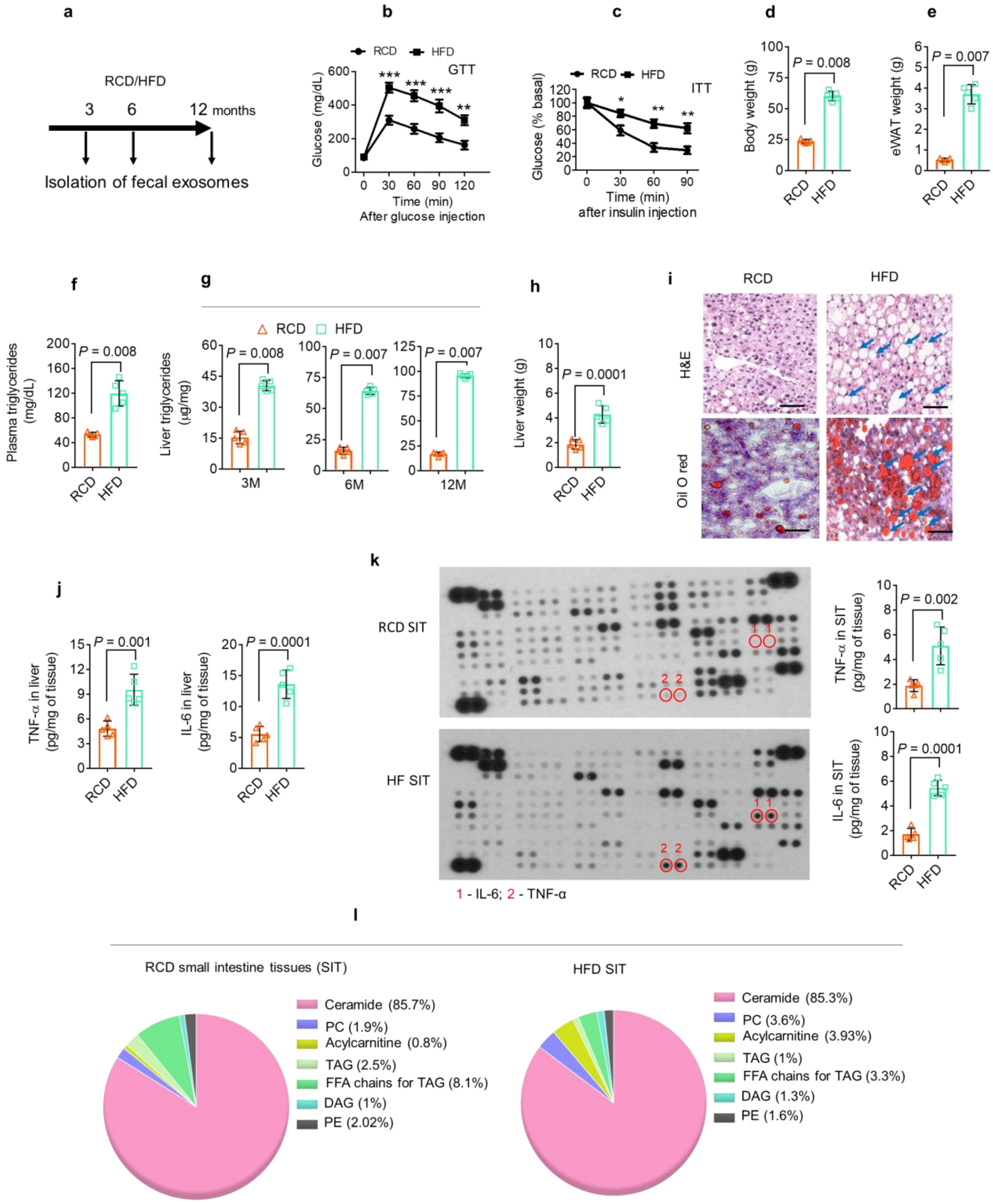
a
RCD/HFD
3
6
12 months
Isolation of fecal exosomes
b
RCD
HFD
GTT
Glucose (mg/dL)
Time (min)
After glucose injection
c
RCD
HFD
ITT
Glucose (% basal)
Time (min)
after insulin injection
d
Body weight (g)
P = 0.008
e
eWAT weight (g)
P = 0.007
f
Plasma triglycerides (mg/dL)
P = 0.008
g
RCD
HFD
Liver triglycerides (µg/mg)
P = 0.008
P = 0.007
P = 0.007
3M
6M
12M
h
Liver weight (g)
P = 0.0001
i
RCD
HFD
H&E
Oil O red
j
TNF-α in liver (pg/mg of tissue)
P = 0.001
IL-6 in liver (pg/mg of tissue)
P = 0.0001
k
RCD SIT
HF SIT
1 - IL-6; 2 - TNF-α
TNF-α in SIT (pg/mg of tissue)
P = 0.002
IL-6 in SIT (pg/mg of tissue)
P = 0.0001
l
RCD small intestine tissues (SIT)
Ceramide (85.7%)
PC (1.9%)
Acylcarnitine (0.8%)
TAG (2.5%)
FFA chains for TAG (8.1%)
DAG (1%)
PE (2.02%)
HFD SIT
Ceramide (85.3%)
PC (3.6%)
Acylcarnitine (3.93%)
TAG (1%)
FFA chains for TAG (3.3%)
DAG (1.3%)
PE (1.6%)

Supplementary Figure 1: Long-term HFD feeding induced obesity, insulin and glucose intolerance, liver and small intestine tissue inflammation with lipid changes.

a. Graphical presentation of the timeline over which mice were fed a regular chow diet (RCD) or high-fat diet (HFD). The downward arrows indicate the times at which exosome isolation from feces was conducted (n = 10 mice/group). **b.** GTT and **c.** ITT of RCD and HFD mice from which fecal exosomes were isolated (n = 10 mice/group). **d.** Body weight (n=5/group), hollow triangle - RCD fed mice and hollow rectangle - HFD fed mice. **e.** eWAT weight (n=5/group). **f.** Plasma triglycerides for 12M (months) HFD mice (n=5/group). **g.** Liver triglycerides (for models 3, 6 & 12M feeding either RCD or HFD) (n=5/group). **h.** Liver weight (n=5/group). **i.** H&E and oil red O staining of liver tissues. Blue arrows show lipid droplets. Scale bar is 20μm. Data is from experiments repeated at least three times. **j.** Inflammatory cytokines (TNF-α & IL-6) levels in liver tissues (n=5/group). **k.** Inflammatory cytokines (TNF-α & IL-6) levels in small intestine tissues (SIT). Quantification of TNF-α & IL-6 shown in right (n=5/group). **l.** Lipidomic analysis of bioactive lipids of SIT of RCD & HFD 12M fed mice. Data are presented as the mean ± SD. Student's *t* test, two-tailed. *<0.05; **<0.01; ***<0.001; ****<0.0001. Source data are provided as a Source Data file.

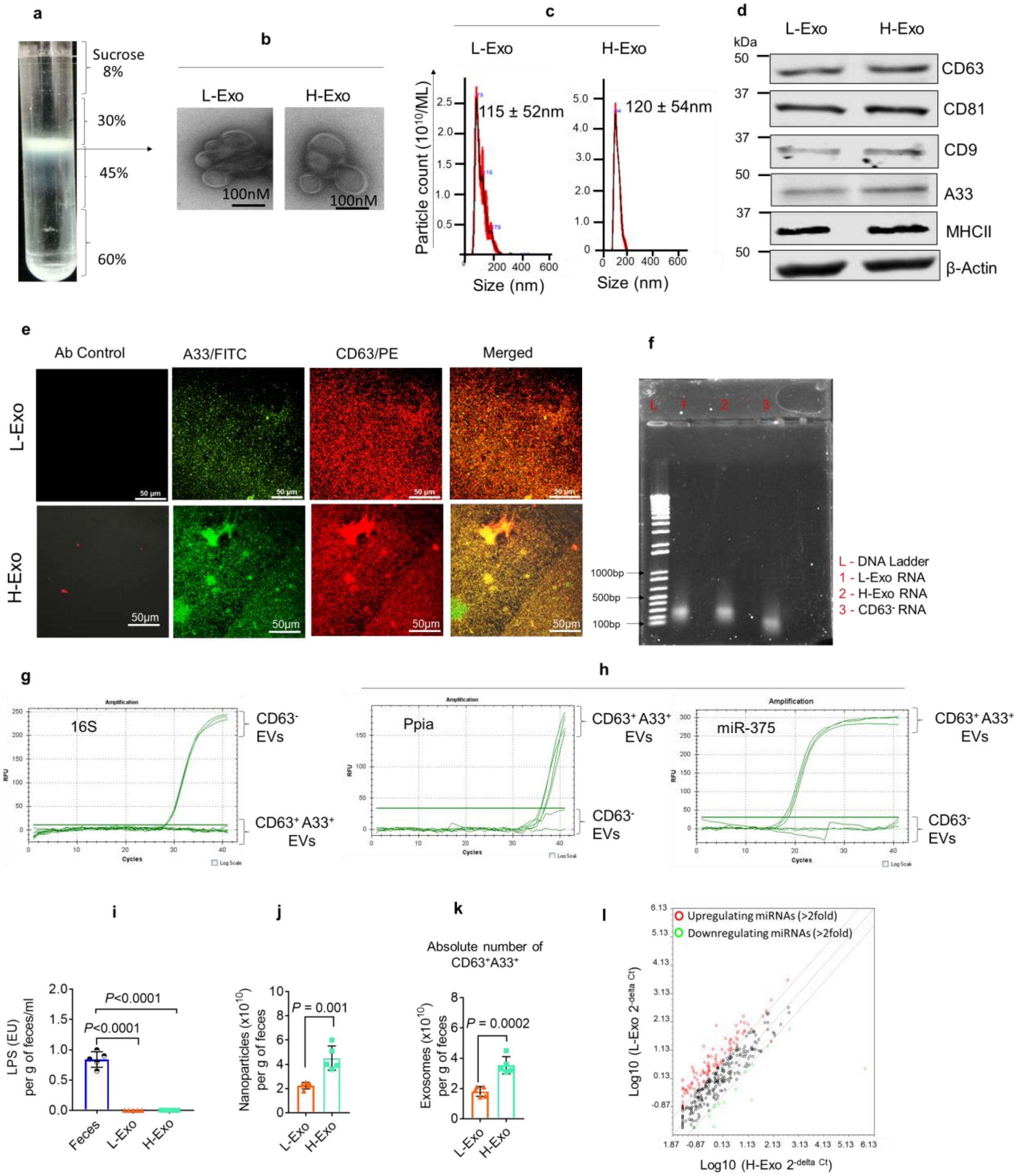

a
Sucrose 8%
30%
45%
60%
b
L-Exo
H-Exo
100nM
100nM
c
L-Exo
H-Exo
Particle count (10^10/ML)
115 ± 52nm
120 ± 54nm
Size (nm)
Size (nm)
d
L-Exo
H-Exo
kDa
CD63
CD81
CD9
A33
MHCII
β-Actin
e
Ab Control
A33/FITC
CD63/PE
Merged
L-Exo
H-Exo
50µm
f
L - DNA Ladder
1 - L-Exo RNA
2 - H-Exo RNA
3 - CD63- RNA
1000bp
500bp
100bp
g
16S
CD63- EVs
CD63+ A33+ EVs
h
Ppia
CD63+ A33+ EVs
CD63- EVs
miR-375
CD63+ A33+ EVs
CD63- EVs
Amplification
Cycles
RFU
i
LPS (EU) per g of feces/ml
P<0.0001
P<0.0001
Feces
L-Exo
H-Exo
j
Nanoparticles (x10^10) per g of feces
P = 0.001
L-Exo
H-Exo
k
Absolute number of CD63+A33+
Exosomes (x10^10) per g of feces
P = 0.0002
L-Exo
H-Exo
l
Upregulating miRNAs (>2fold)
Downregulating miRNAs (>2fold)
Log10 (L-Exo 2-delta Ct)
Log10 (H-Exo 2-delta Ct)

Supplementary Figure 2: Characterization of mouse intestinal epithelial cells (IEC) released exosomes.

a. Representative image of sucrose gradient purification of H-Exo. The band at the interphase of 30% & 45% sucrose was used in downstream analysis. **b.** Electron microscopic images of exosomes from lean RCD mice (L-Exo) and HFD mice (H-Exo). Data is from experiments repeated at least three times. **c.** The exosome size was estimated using a Malvern NanoSight NS300 (Malvern Instruments). Data is from experiments repeated at least three times. **d.** Western blot images showing expression of CD63, CD81, CD9 (exosome marker) and A33 & MHCII (intestinal epithelial cell marker) on both L-Exo and H-Exo exosomes. Data is from experiments repeated at least three times. **e.** Exosomes were stained with A33 (green/FITC) and CD63 (red/PE) antibodies. Exosomes were analyzed by confocal microscopy. Scale bar as indicated. Data is from experiments repeated at least three times. **f.** Agarose gel profile for RNA extracted from $CD63^{+}A33^{+}$ and $CD63^{-}$ EVs. Data from experiments repeated at least three times. **g.** qPCR amplification graph for 16S. **h.** Cyclophilin A (*Ppia*) and miR-375 with cDNA prepared from $CD63^{+}A33^{+}$ and $CD63^{-}$EVs RNA. **i.** LPS detection in pulled down $CD63^{+}A33^{+}$ exosomes (n=5/group). Half-filled circle - feces, filled triangle - L-Exo and filled rectangle - H-Exo. **j.** Nanoparticle yield per gram of mouse feces was determined using the NS300 (n=5/group). **k.** Absolute number of $CD63^{+}A33^{+}$ exosomes after pull-down was determined using the NS300 (n=5/group). **l.** Scatter plot showing levels of miRNA in L-Exo vs H-Exo. Data are presented as the mean ± SD. Student's *t* test (two-tailed) and one-way ANOVA with Tukey post hoc test. Source data are provided as a Source Data file.

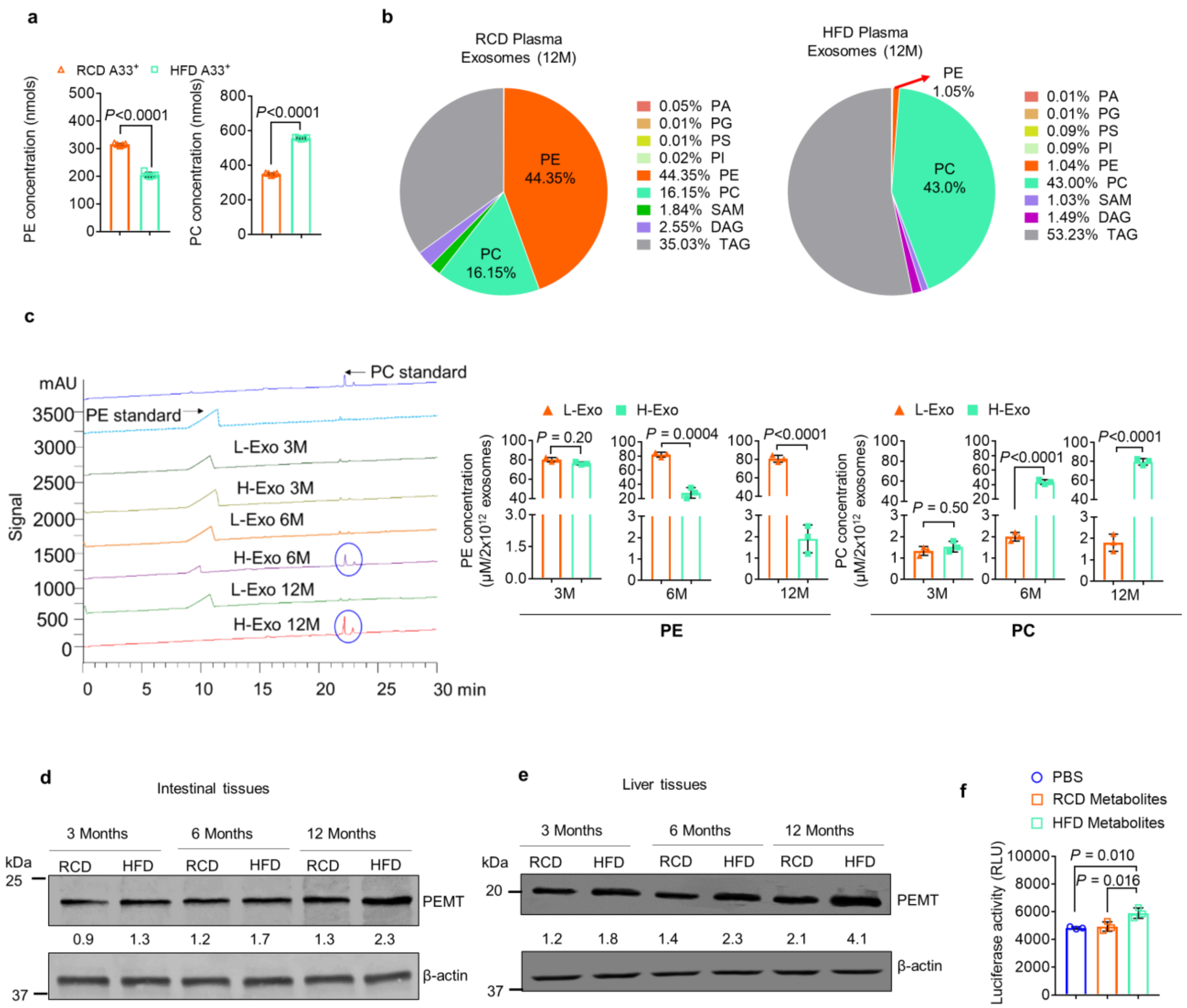

Supplementary Figure 3: HFD led to increase the PC in plasma exosomes and intestinal epithelial cells (A33$^+$).

a. Phosphatidylethanolamine (PE) and Phosphatidylcholine (PC) concentration (nmols) determined by triple quadrupole MS in FACS sorted A33$^+$ intestinal epithelial cells derived from mice (n=5/group) fed either a regular chow diet (RCD) or a high-fat diet (HFD) for 12 months. **b.** Lipids were extracted from plasma exosomes of mice fed either a RCD or a HFD for 12 months (n=10/group). Pie charts representing the percentage of each lipid, as determined using triple quadrupole mass spectrometry. **c.** Representative HPLC analysis of PE and PC lipids in mouse intestinal epithelial exosomes via detection of UV absorbance at 220 nm. PE and PC concentration shown at right (n=3). Filled triangle - L-Exo and filled rectangle - H-Exo. **d.** & **e.** immunoblots showing PEMT protein levels in mouse intestinal (**d**) and liver (**e**) tissue extracts after mice were fed either a RCD or HFD for 3, 6, or 12 months. Ratio to β-actin shown in the middle as numbers. Data is from experiments repeated at least three times. **f.** Mouse colon (MC-38) cells were transfected with pGL3B-PEMT-luc and treated with fecal metabolites from mice fed either a RCD or HFD for 12 months. Normalized luciferase activity was measured as an indication of

PEMT expression (n=3/group). Data are represented as mean ± S.D. Student's *t* test (two-tailed) or one-way ANOVA with a Tukey post hoc test. Source data are provided as a Source Data file.

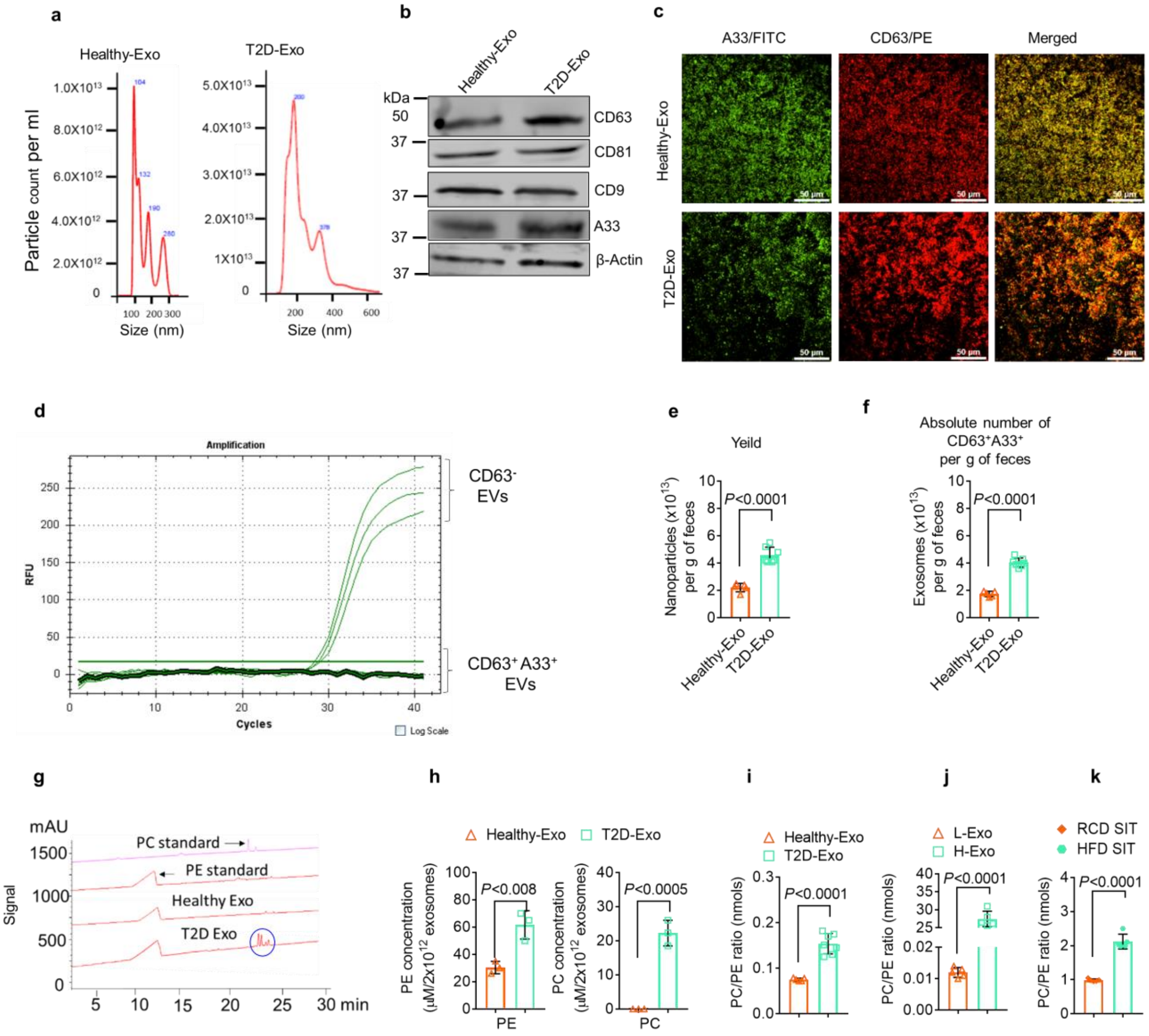

Supplementary Figure 4: Characterization of human IEC released ($CD63^+A33^+$) exosomes.

a. The size of fecal exosomes isolated from healthy control (Healthy-Exo) and type 2 diabetic (T2D-Exo) humans was estimated using the NS300. **b.** Western blot images confirming expression of CD63, CD81, CD9 and A33 in human fecal exosomes. Data is from experiments repeated at least three times. **c.** Healthy control and T2D patient-derived exosomes were stained with A33 (green/FITC) and CD63 (red/PE) antibodies and analyzed using confocal microscopy. Scale bar as indicated. Data is from experiments repeated at least three times. **d.** qPCR amplification for 16S with cDNA prepared from $CD63^+A33^+$ and $CD63^-$ EV RNA derived from human feces. **e.** Exosome yield was determined per gram of healthy individuals (n=5) and T2D patients (n=7) feces. Hollow triangle - Healthy-Exo and hollow rectangle - T2D-Exo. **f.** Absolute number of

exosomes after pull-down by CD63 and A33 (Healthy-Exo=5; T2D-Exo=7). **g.** HPLC analysis of PE and PC in human fecal exosomes from T2D patients and healthy controls. PE and PC concentration determined by HPLC (n=3/group). **i-k.** PC/PE ratio calculated using the data from triple quadrupole MS in human exosomes (Healthy-Exo=5; T2D-Exo=7; **i**), 12M mice (n=5) exosome (**j**) and small intestinal tissue (SIT; **k**). Filled diamond – RCD SIT and filled hexagon – HFD SIT. Data are presented as the mean ± SD. Student's *t* test, two-tailed. Source data are provided as a Source Data file.

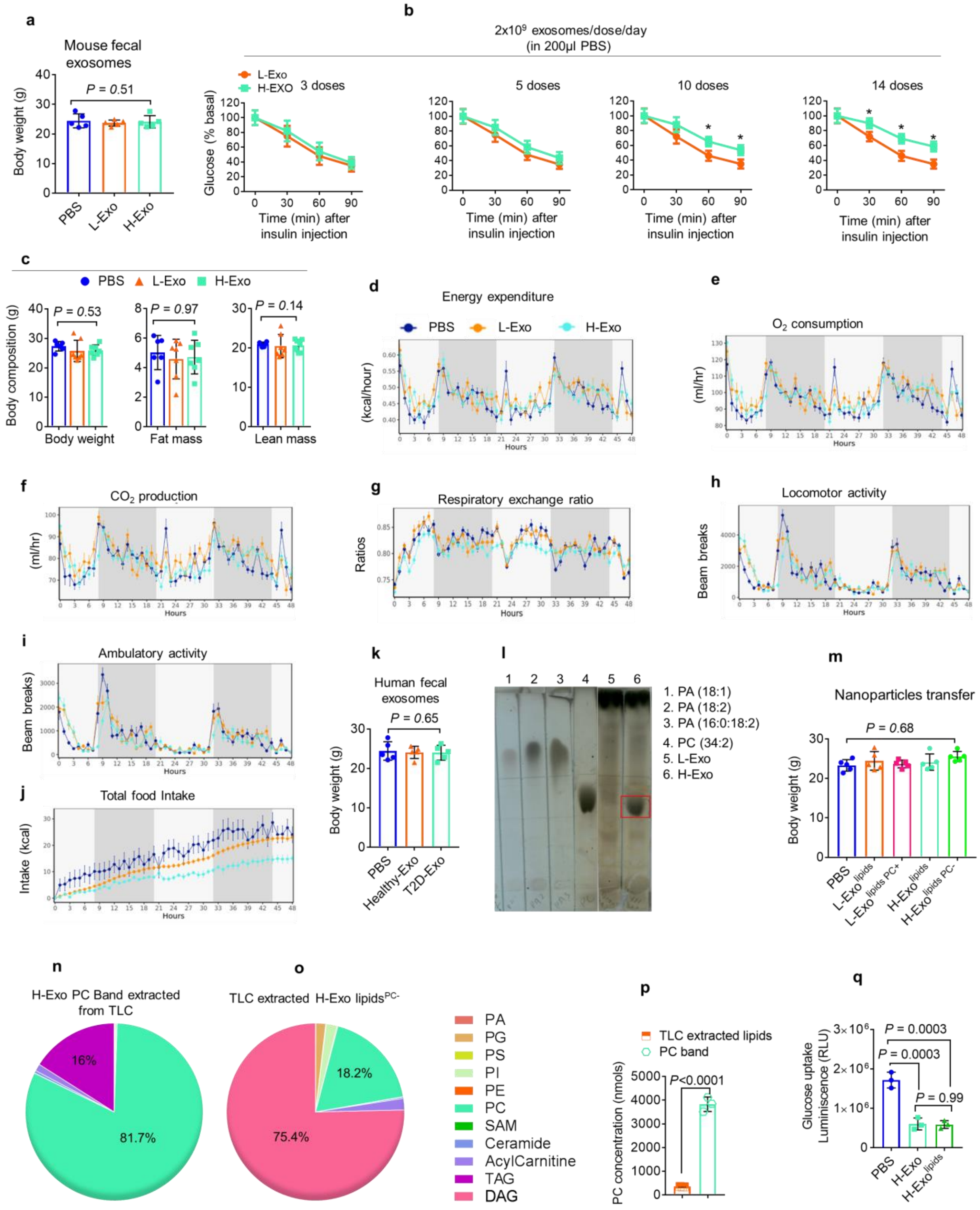
a
Mouse fecal exosomes
P = 0.51
Body weight (g)
PBS
L-Exo
H-Exo
b
2x10^9 exosomes/dose/day
(in 200µl PBS)
L-Exo
H-EXO
3 doses
5 doses
10 doses
14 doses
Glucose (% basal)
Time (min) after insulin injection
c
PBS L-Exo H-Exo
P = 0.53
P = 0.97
P = 0.14
Body composition (g)
Body weight
Fat mass
Lean mass
d
Energy expenditure
PBS L-Exo H-Exo
(kcal/hour)
e
O2 consumption
(ml/hr)
f
CO2 production
(ml/hr)
g
Respiratory exchange ratio
Ratios
h
Locomotor activity
Beam breaks
i
Ambulatory activity
Beam breaks)
j
Total food Intake
Intake (kcal)
Hours
k
Human fecal exosomes
P = 0.65
Body weight (g)
PBS
Healthy-Exo
T2D-Exo
l
1. PA (18:1)
2. PA (18:2)
3. PA (16:0:18:2)
4. PC (34:2)
5. L-Exo
6. H-Exo
m
Nanoparticles transfer
P = 0.68
Body weight (g)
PBS
L-Exo lipids
L-Exo lipids PC+
H-Exo lipids
H-Exo lipids PC-
n
H-Exo PC Band extracted from TLC
16%
81.7%
o
TLC extracted H-Exo lipids PC-
18.2%
75.4%
PA
PG
PS
PI
PE
PC
SAM
Ceramide
AcylCarnitine
TAG
DAG
p
TLC extracted lipids
PC band
P<0.0001
PC concentration (nmols)
q
P = 0.0003
P = 0.0003
P = 0.99
Glucose uptake Luminiscence (RLU)
PBS
H-Exo
H-Exo lipids

Supplementary Figure 5: H-Exo oral administration has no impact on mice physiology other than glucose and insulin intolerance.

a. Mice body weight after receiving PBS, L-Exo or H-Exo for 14 days while fed HFD. Filled circle – PBS; filled triangle - L-Exo and filled rectangle - H-Exo. **b.** H-Exo dose dependent impairment in insulin response in mice (n=5/group). **c.** Body composition (body weight, lean and fat mass) determined by NMR prior performing clamp assay (n=6/group). **d. – j.** Parameters associated with mouse physiology (energy expenditure, O_2 consumption, CO_2 production, respiratory exchange ratios, locomotor & ambulatory activities and food intake) assessed by metabolic cages (CLAMS). **k.** Body weight of mice receiving Healthy-Exo or T2D-Exo for 14 days while fed HFD (n=5/group). **l.** Thin layer chromatography (TLC) of total lipids from L-Exo or H-Exo indicating PC band along with standard PA and PC. **m.** Mice body weight after receiving nanoparticles (generated from total lipids from L-Exo or H-Exo and PC depleted or added lipids) for 14 days while fed HFD (n=5/group). **n.** Pie charts of lipids from PC band (PCB; depleted as shown red rectangle in panel **l**). **o.** Pie charts of lipids quantified by quadrupole MS of remainder lipids (TLC extracted H-Exo lipids^{PC-}). **p.** Quantitative analysis of PC concentration in TLC extracted lipids after PC band depletion; PC band analyzed by triple quadrupole MS (n=3). **q.** Glucose uptake by FL83B cells treated (n=3) with H-Exo or its total lipids (H-Exo^{nano}). Data are presented as the mean ± SD. One-way ANOVA with a Bonferroni or Tukey post hoc test and Student’s *t* test, two-tailed. Source data are provided as a Source Data file.

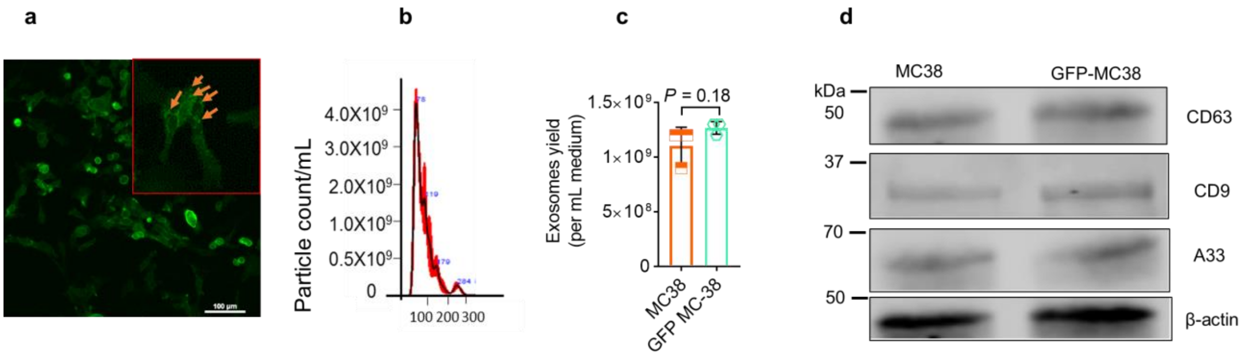

Supplementary Figure 6: Characterization of GFP-MC38 released exosomes.
a. Live cell imaging of GFP-MC38 cells releasing GFP positive exosomes shown by orange arrows (inset). Scale bar as indicated. Data is from experiments repeated at least three times. **b.** & **c.** Size (**b**) and yield (**c**) estimation of exosomes from culture supernatant of WT MC38 and GFP-MC38 cells as assessed using the NS300 (n=3). **d.** Western blot analysis of CD63, CD9 and A33 expression on WT MC38 and GFP-MC38-released exosomes**.** Data is from experiments repeated at least three times. Data are presented as the mean ± SD. Student's *t* test, two-tailed. Source data are provided as a Source Data file.

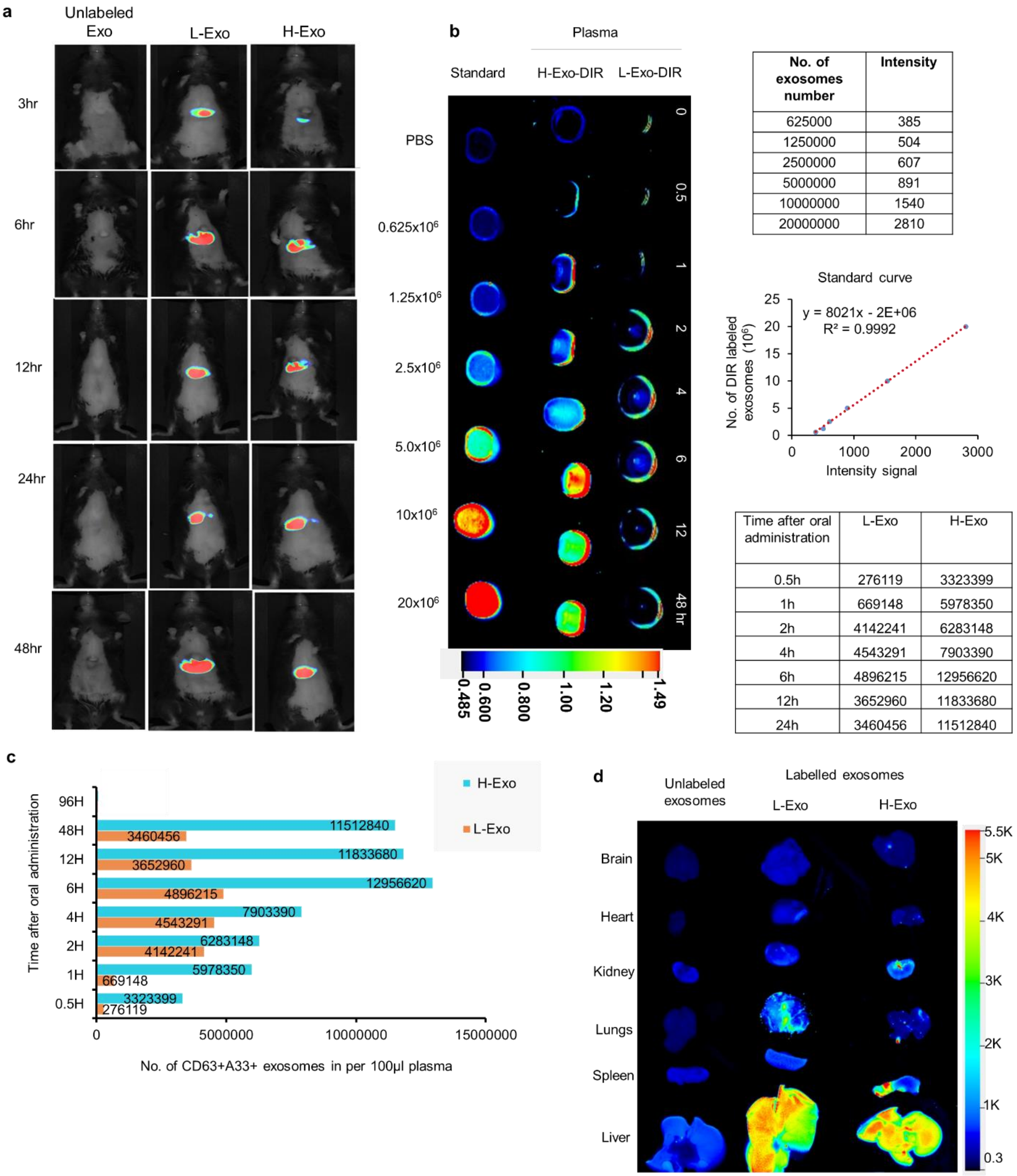

No. of exosomes number	Intensity
625000	385
1250000	504
2500000	607
5000000	891
10000000	1540
20000000	2810

Time after oral administration	L-Exo	H-Exo
0.5h	276119	3323399
1h	669148	5978350
2h	4142241	6283148
4h	4543291	7903390
6h	4896215	12956620
12h	3652960	11833680
24h	3460456	11512840

Supplementary Figure 7: CD63$^+$A33$^+$ exosomes preferential targets the liver via circulation.

a. Live imaging of mice orally gavaged with DIR-labelled exosomes at different time intervals. **b.** DIR labeled CD63$^+$A33$^+$ exosomes detected in plasma at different time points. Standard curve was prepared with known numbers of DIR labeled exosomes. **c.** Numbers of exosomes were calculated with signal intensity in plasma. **d.** Scanned images of preferential localization of both L-Exo and H-Exo to the liver. Data is from experiments repeated at least three times.

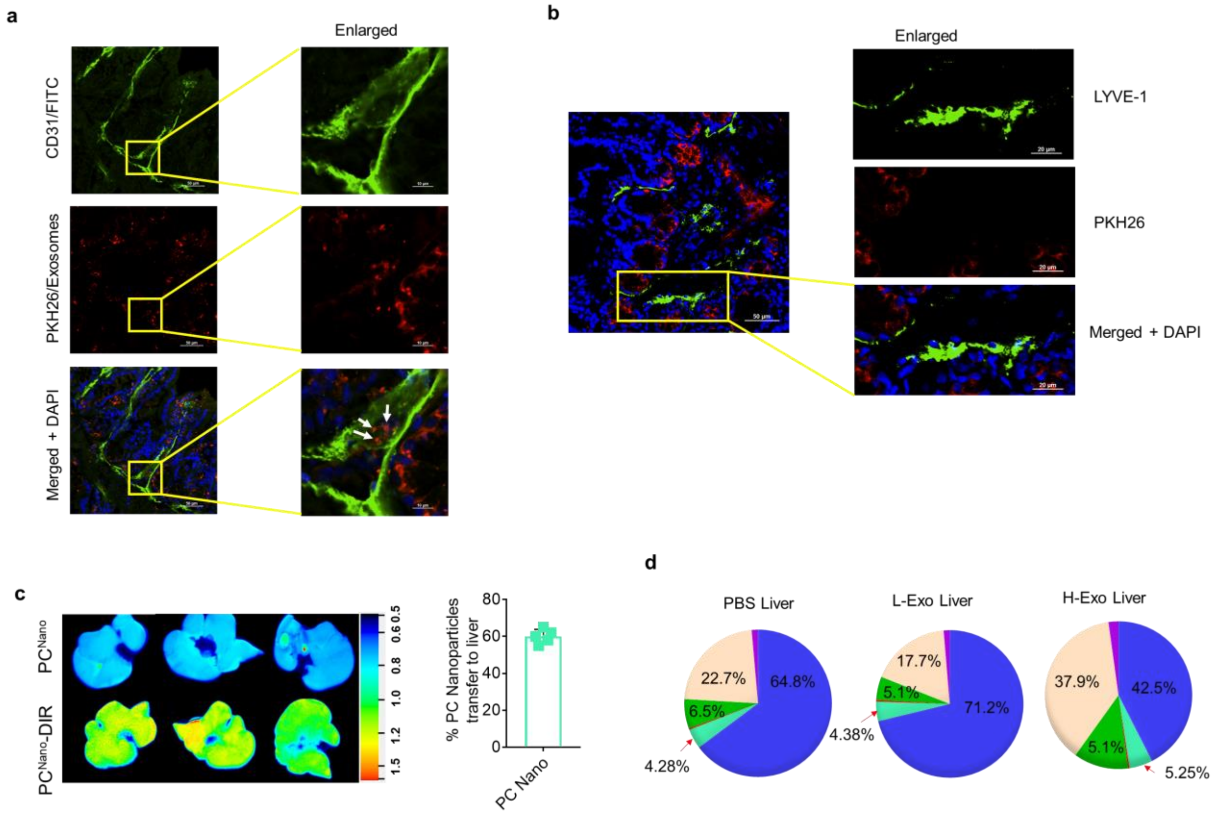

Supplementary Figure 8: Exosomes trafficked from gut to liver via the portal vein but not through the lymphatic circulation.

a. – **b.** Confocal image analysis of frozen sections of intestines from mice orally gavaged with PKH-26 labeled exosomes ($2x10^9$) after immunofluorescent staining for CD31 (**a**; white arrows) and LYVE-1 (**b**) with enlargement of indicated area shown in the right panel. Scale bar as indicated. Data is from experiments repeated at least three times. **c.** Detection of fluorescent (DIR) PC (34:2) nanoparticles in liver. Percentages shown at right (n=5). **d.** Pie charts representing the targeted lipidomics of liver tissue from PBS, L-Exo or H-Exo recipient mice for 14 days. Data are represented as mean ± SD. Data is from experiments repeated at least three times.

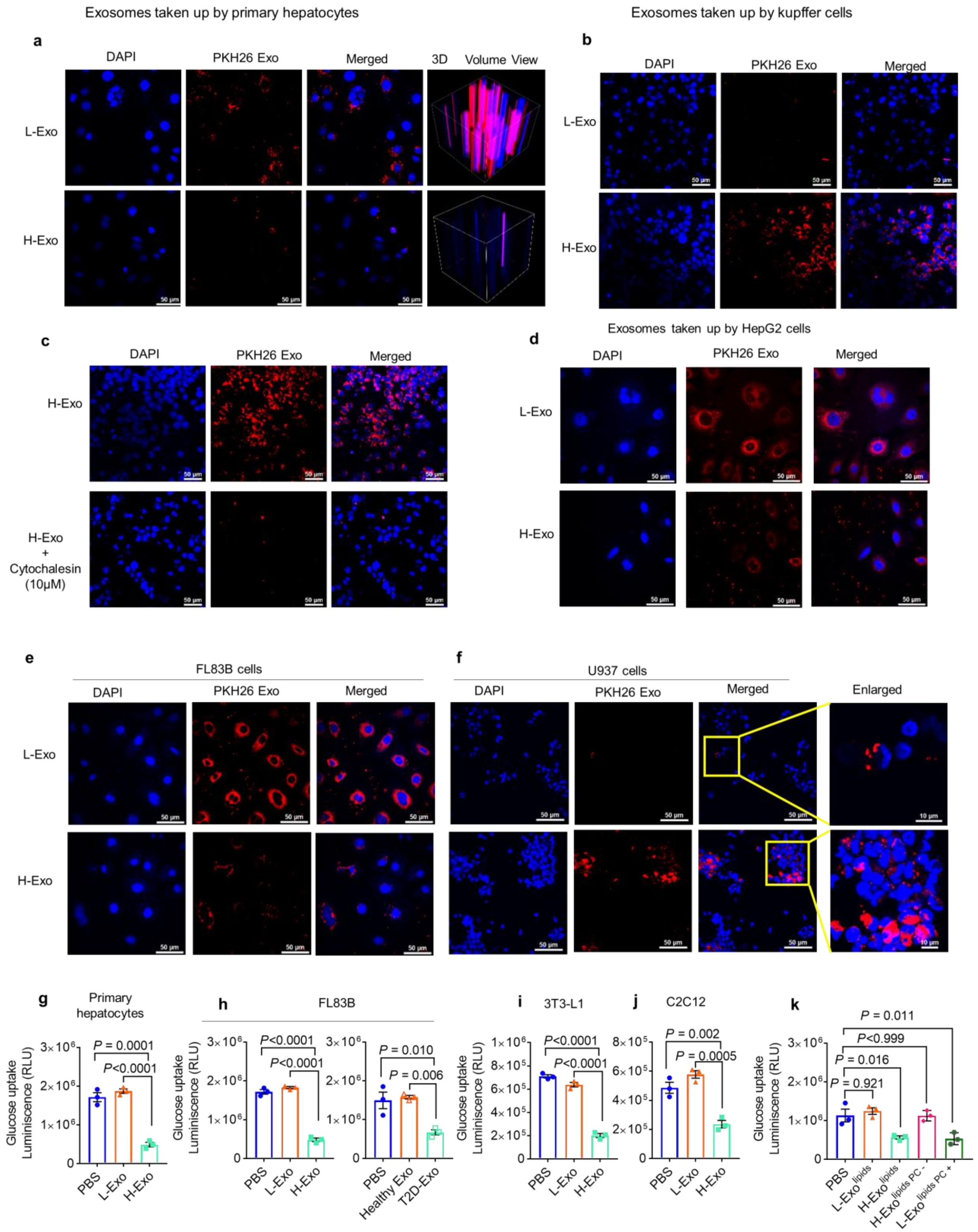
Exosomes taken up by primary hepatocytes
a
DAPI
PKH26 Exo
Merged
3D Volume View
L-Exo
H-Exo
Exosomes taken up by kupffer cells
b
DAPI
PKH26 Exo
Merged
L-Exo
H-Exo
c
DAPI
PKH26 Exo
Merged
H-Exo
H-Exo + Cytochalesin (10μM)
Exosomes taken up by HepG2 cells
d
DAPI
PKH26 Exo
Merged
L-Exo
H-Exo
e
FL83B cells
DAPI
PKH26 Exo
Merged
L-Exo
H-Exo
f
U937 cells
DAPI
PKH26 Exo
Merged
Enlarged
g
Primary hepatocytes
Glucose uptake Luminiscence (RLU)
P = 0.0001
P<0.0001
PBS
L-Exo
H-Exo
h
FL83B
P<0.0001
P<0.0001
P = 0.010
P = 0.006
PBS
Healthy Exo
T2D-Exo
i
3T3-L1
P<0.0001
P<0.0001
j
C2C12
P = 0.002
P = 0.0005
k
P = 0.011
P<0.999
P = 0.016
P = 0.921
L-Exo lipids
H-Exo lipids
H-Exo lipids PC -
L-Exo lipids PC +

Supplementary Figure 9: Liver cells uptake of L-Exo versus H-Exo.

a. Visualization of in vitro uptake of PKH26-labelled L-Exo or H-Exo by mouse primary hepatocytes with accompanying 3D images. Scale bar as indicated. **b.** In vitro uptake of PKH26-labeled L-Exo or H-Exo by mouse Kupffer cells. **c.** Inhibition of phagocytosis by cytochalasin D in mice Kupffer cells. **d.** – **f.** Uptake of PKH26-labelled L-Exo or H-Exo by human hepatocytes (HepG2 cells; **d**), mouse hepatocyte (FL83B; **e**) and human monocyte cell (U937; **f**) lines. **g.** Glucose uptake assays performed on mouse primary hepatocytes cultured with PBS and $CD63^{+}A33^{+}$ mouse exosomes (L-Exo and H-Exo) for 16 hours (n=3). Filled circle – PBS; filled triangle - L-Exo and filled rectangle - H-Exo. **h.** - **j.** Glucose uptake assay of mouse hepatocytes (FL83B; **h**), adipocyte (3T3-L1; **i**) and skeletal muscle (C2C12; **j**) cell lines cultured with CD63+A33+ exosomes for 16 hours (n=3). Hollow triangle - Healthy-Exo and hollow rectangle - H-Exo. **k.** Nanoparticles generated from total lipids, PC depleted & added lipids of $CD63^{+}A33^{+}$ exosomes. Glucose uptake assay of mouse primary hepatocytes cultured with nanoparticles for 16 hours (n=3). Data are represented as mean ± SD. One-way ANOVA with a Tukey post hoc test. Source data are provided as a Source Data file. Data is from experiments repeated at least three times.

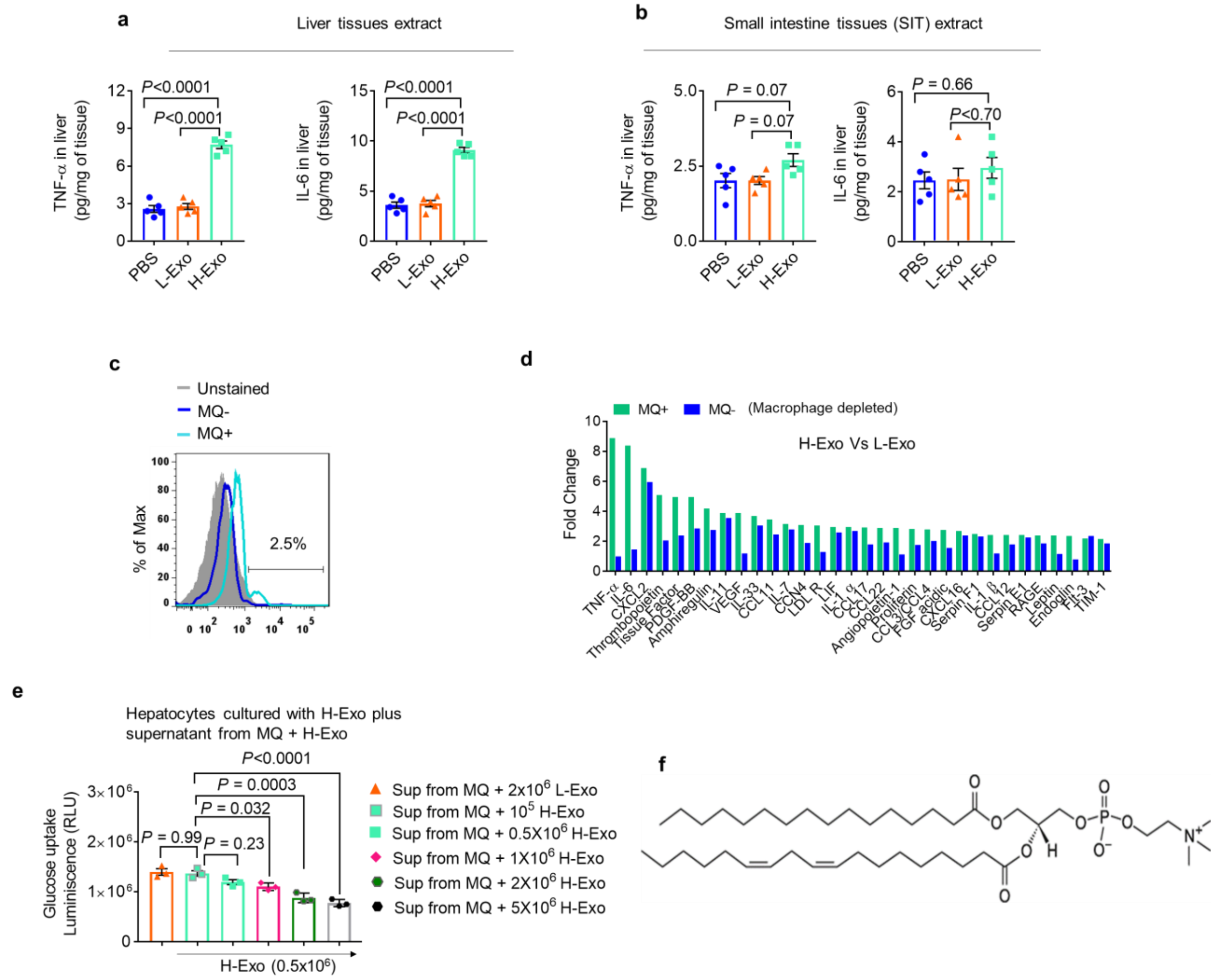

Supplementary Figure 10: H-Exo induce inflammation in liver and WAT but not in intestinal tissues of recipient mice.

a. – **b.** Inflammatory cytokines (TNF-α & IL-6) in liver (**a**) and in small intestine tissues (**b**) quantified by ELISA (n=5). Filled circle – PBS; filled triangle - L-Exo and filled rectangle - H-Exo. **c.** Macrophage (MQ) depletion by a single injection of clodrosomes^{R} as assessed by flow cytometry for whole blood staining of F4/80. **d.** Fold change in H-Exo vs L-Exo-induced cytokines levels in mouse plasma with or without macrophage depletion. **e.** Glucose uptake assay (n=3) performed on mouse hepatocytes supplemented with supernatant derived from macrophages cultured with nanoparticles derived from H-Exo total lipids (H-Exo Nano) and PC (34:2). **f.** Fatty acid structure of PC (34:2) purchased from Avanti Polar Lipids Inc. Data are represented as mean ± SD. One-way ANOVA with a Tukey post hoc test. Source data are provided as a Source Data file.

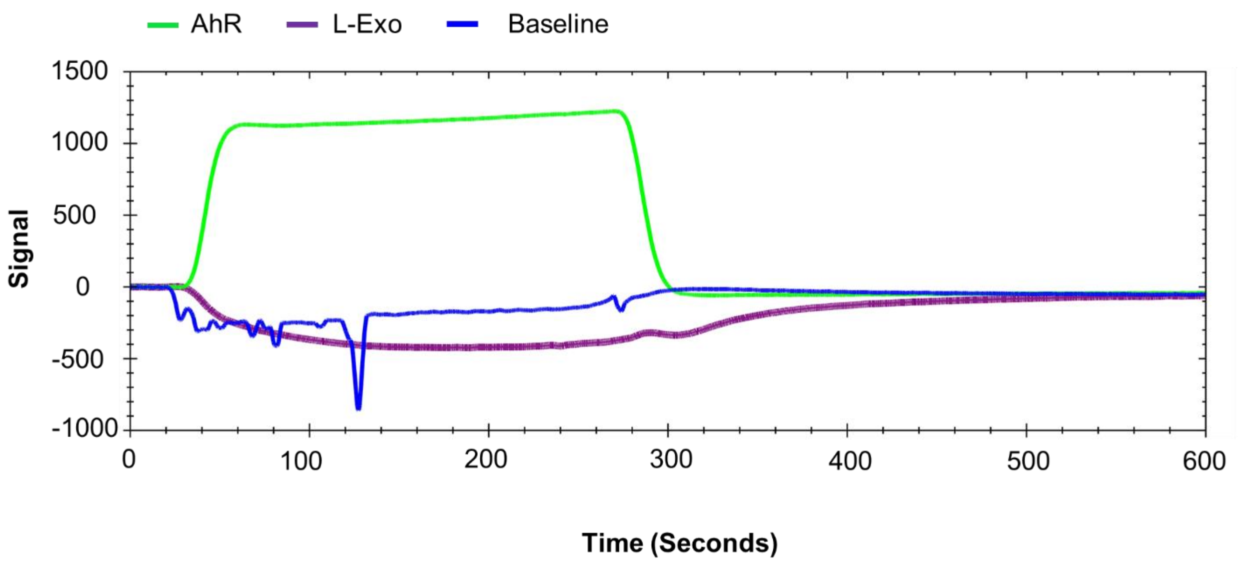

Supplementary Figure 11: SPR sensogram showing no interaction of AhR with L-Exo.

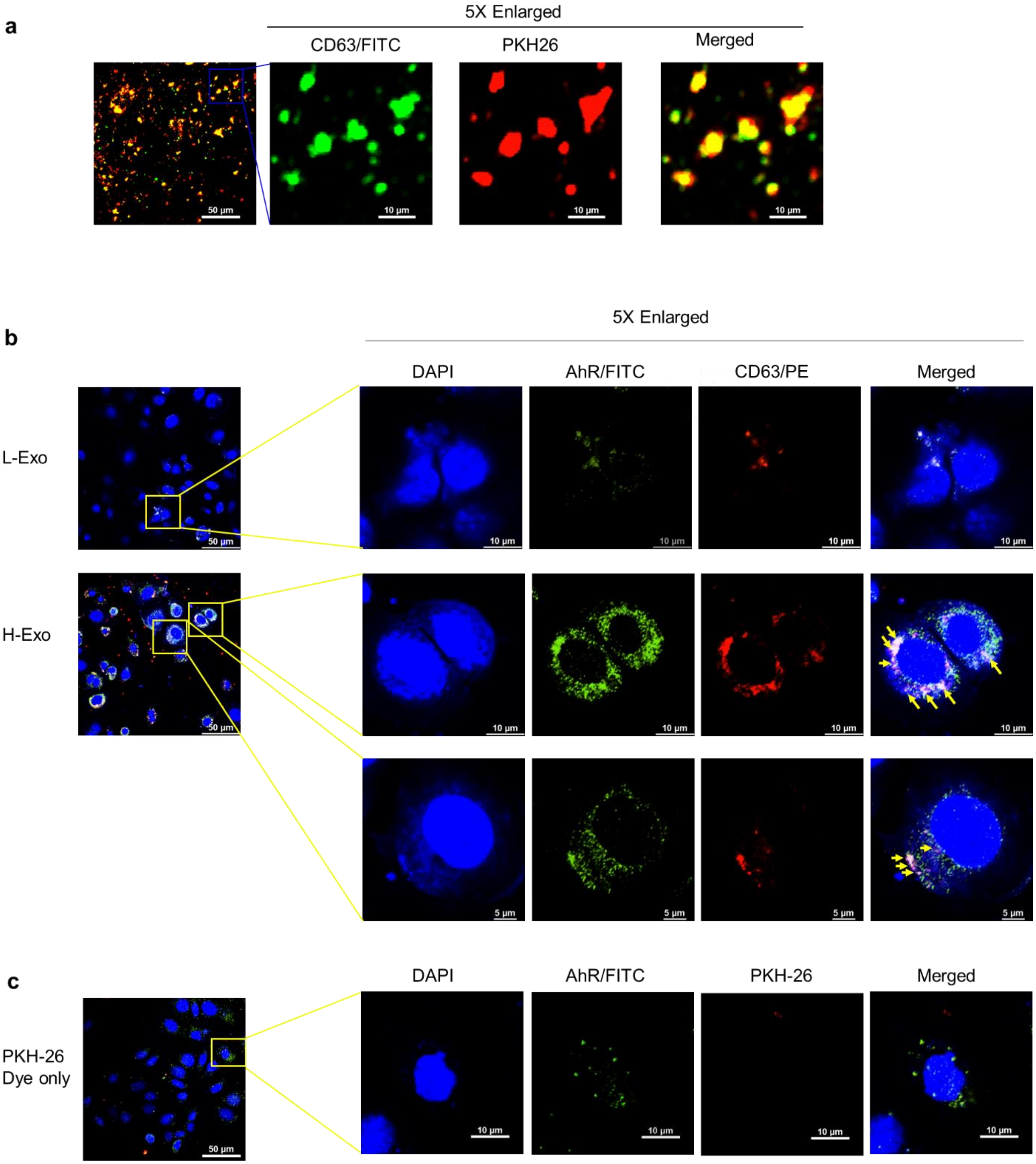
a
5X Enlarged
CD63/FITC
PKH26
Merged
50 µm
10 µm
b
5X Enlarged
DAPI
AhR/FITC
CD63/PE
Merged
L-Exo
H-Exo
5 µm
c
DAPI
AhR/FITC
PKH-26
Merged
PKH-26
Dye only

Supplementary Figure 12: Co-location of PKH-26 dye signal with exosomal marker on H-Exo.

a. PKH-26 dye signals co-localized with the CD63 exosomal marker on H-Exo. Scale bar as indicated. **b.** L-Exo or H-Exo interaction with AhR in FL83B cells as indicated by the yellow arrows. Scale bar as indicated. **c.** Only PKH-26 dye in the cell culture. Scale bar as indicated. Data is from experiments repeated at least three times.

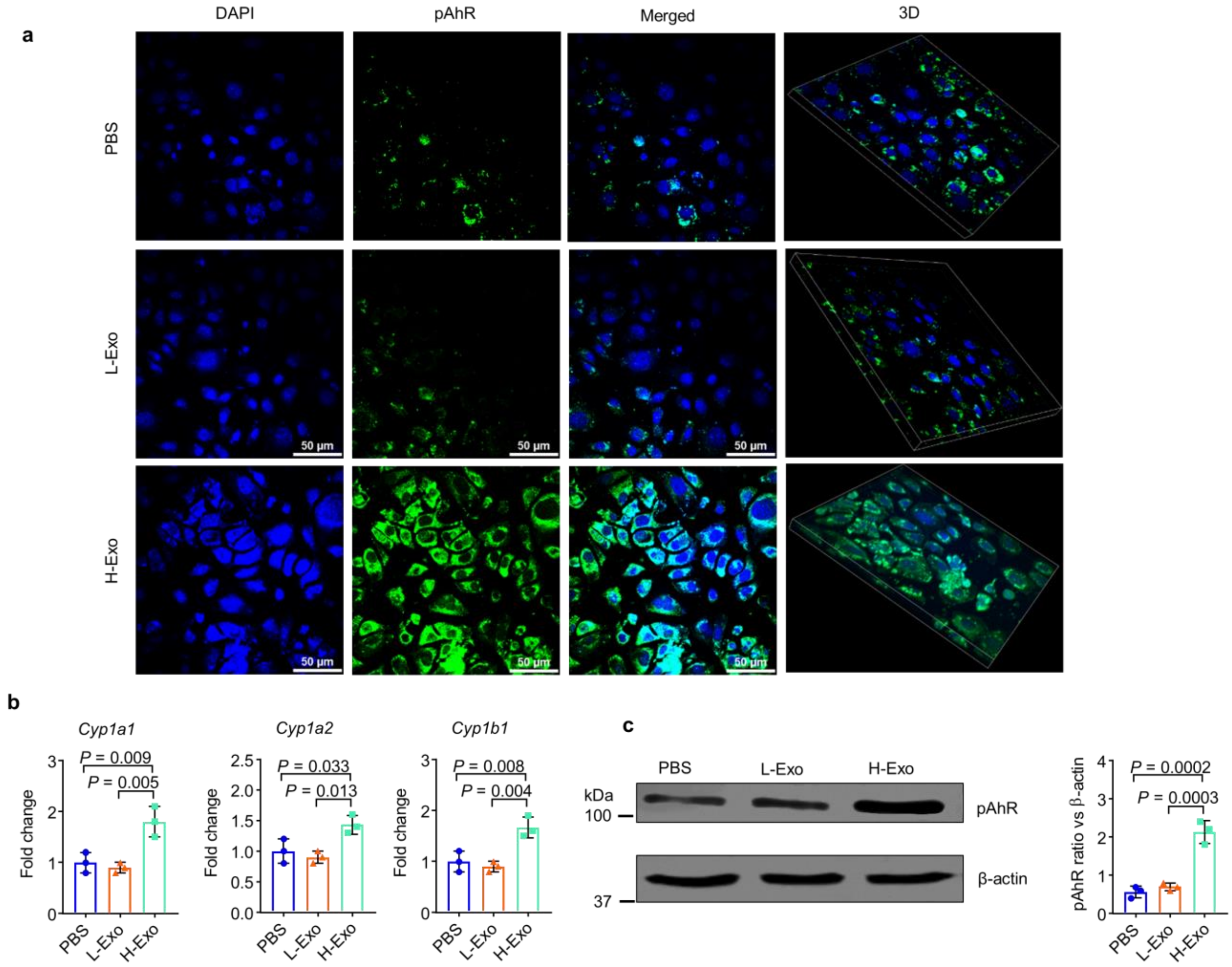

Supplementary Figure 13: H-Exo induced pAhR expression in hepatocytes.

a. Mouse hepatocytes (FL83B cells) were cultured with L-Exo and H-Exo for 16 hours and induction of pAhR in the nucleus was visualized by confocal microscopy. 3D image shown at right. Scale bar as indicated. **b.** qPCR of AhR downstream genes *Cyp1a1*, *Cyp1a2* and *Cyp1b1* (n=3). Filled circle – PBS; filled triangle - L-Exo and filled rectangle - H-Exo. **c.** Depicting the western blot images of pAhR expression in primary hepatocytes cultured with H-Exo for 16 hours. Ratio to β-actin shown in the right (n=3). Data are represented as mean ± SD. One-way ANOVA with a Tukey post hoc test. Source data are provided as a Source Data file. Data is from experiments repeated at least three times.

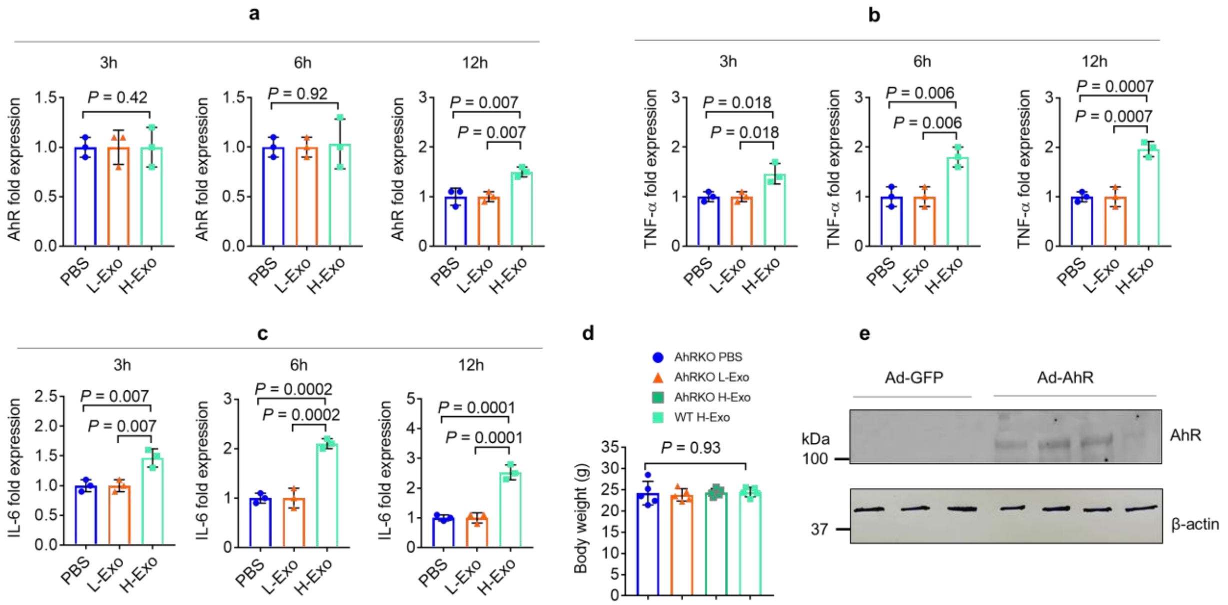

Supplementary Figure 14: H-Exo mediated alteration of inflammatory cytokines gene expression in macrophages.

a. – **c.** qPCR analysis of *AhR* (**a**), *TNF-α* (**b**) and *IL-6* (**c**) gene in Kupffer cells cultured with PBS, L-Exo or H-Exo for 3h, 6h and 12h (n=3). Filled circle – PBS; filled triangle - L-Exo and filled rectangle - H-Exo. **d.** Body weight of *AhR*$^{-/-}$ mice receiving PBS, L-Exo and H-Exo for 14 days while fed HFD (n=5). **e.** Re-expression of AhR in hepatocyte of *AhR*$^{-/-}$ mice by adenovirus injected (5x10^{9} pfu) via the tail vein was confirmed by western blot. Data is from experiments repeated at least three times. Data are represented as mean ± SD. One-way ANOVA with a Tukey post hoc test. Source data are provided as a Source Data file.

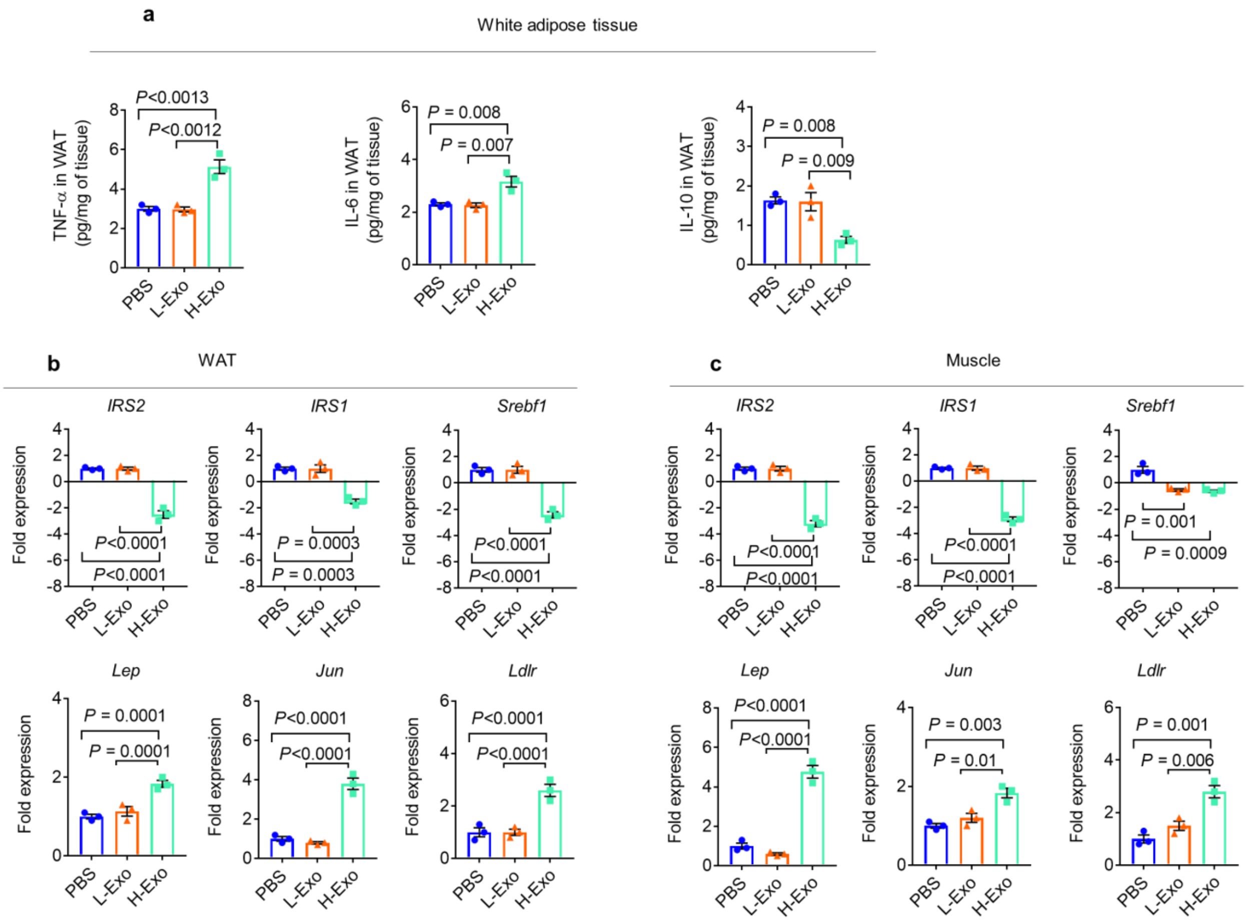

Supplementary Figure 15: H-Exo mediated alteration the expression of cytokines and genes in WAT and muscle tissue.

a. TNF-α, IL-6 and IL-10 measured by ELISA in WAT extracts derived from mice treated with PBS, L-Exo or H-Exo (n=3). Filled circle – PBS; filled triangle - L-Exo and filled rectangle - H-Exo. **b.** & **c.** Up- or downregulated genes in WAT (**b**) and muscle tissues (**c)** derived from mice treated with PBS, L-Exo, and H-Exo for 14 days (n=3). Data are presented as the mean ± SD. One-way ANOVA with a Tukey post hoc test.; Source data are provided as a Source Data file.

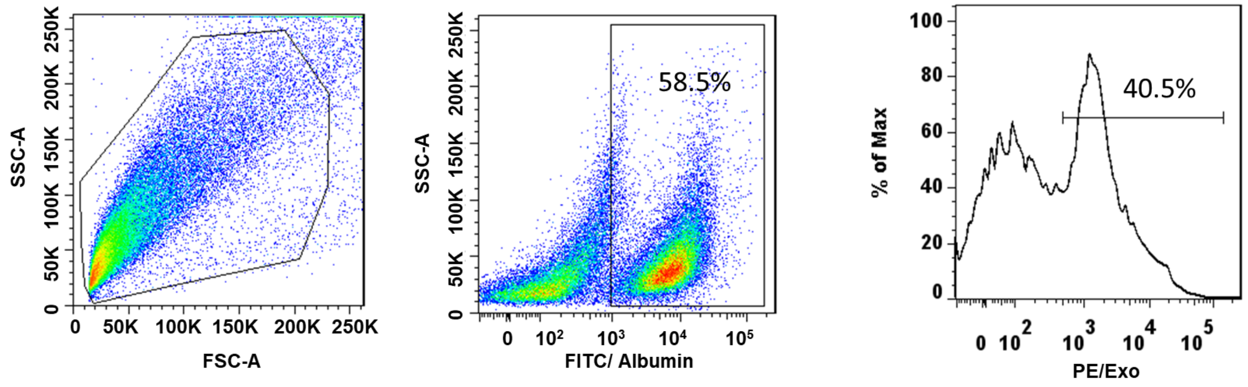

Supplementary Figure 16: Gating strategy for flow cytometric analysis of exosome uptake by liver cells as shown in Figure 3.

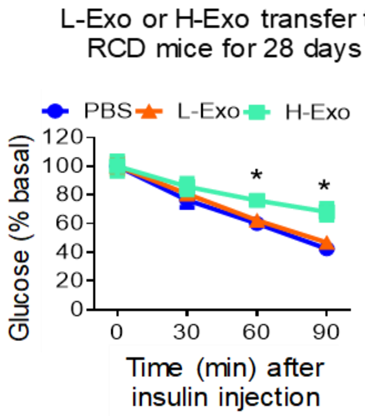

Supplementary Figure 17: Insulin tolerance test of mice that received adoptive transfer of exosomes for 28 days while being fed the regular chow diet Filled circle – PBS; filled triangle - L-Exo and filled rectangle - H-Exo.

Supplementary Table 1. MS/MS analysis of the proteins present on the exosomes derived from RCD (L-Exo) or HFD mice (H-Exo). Data represented as fold change of the signals detected by MS/MS.

S. No.	Identified Proteins	Gene Name (Uniprot)	L-Exo (Signal) n=5	H-Exo (Signal) n=5	H-Exo Vs L-Exo
1	Ig alpha chain C region OS		1E+08	7E+07	0.49
2	Intelectin-1a OS	Itln1	1E+08	158430	0.00
3	Zymogen granule membrane protein 16 OS	Zg16	1E+08	3E+08	2.09
4	Annexin A4 OS	Anxa4	7E+07	4E+06	0.06
5	Isoform 2 of Deleted in malignant brain tumors 1 protein OS	Dmbt1	5E+07	2E+07	0.45
6	Pancreatic triacylglycerol lipase OS	Pnlip	4E+07	22169	0.00
7	Calcium-activated chloride channel regulator 1 OS	Clca1	4E+07	4E+07	1.15
8	Aminopeptidase N OS	Anpep	3E+07	60512	0.00
9	Polymeric immunoglobulin receptor OS	Pigr	2E+07	1E+07	0.54
10	Colipase OS	Clps	2E+07	367920	0.02
11	Protein LEG1 homolog OS	Leg1	2E+07	3E+06	0.17
12	Cluster of Actin, cytoplasmic 1 OS	Actb	1E+07	70181	0.00
13	Isoform 2 of Carcinoembryonic antigen-related cell adhesion molecule 1 OS	Ceacam1	1E+07	6E+06	0.65
14	Cadherin-related family member 2 OS	Cdhr2	9E+06	3E+06	0.31
15	Pancreatic secretory granule membrane major glycoprotein GP2 OS	Gp2	6E+06	2E+06	0.26
16	N-acetylated-alpha-linked acidic dipeptidase-like protein OS	Naaladl1	4E+06	4E+06	1.18
17	Mucin-2 (Fragments) OS	Muc2	3E+06	7E+06	2.56
18	Histone H4 OS	Hist1h4a	2E+06	0	0.00
19	Sodium/potassium-transporting ATPase subunit alpha-2 OS	Atp1a2	351190	0	0.00
20	Cluster of Serine protease inhibitor A3K OS	Serpina3k	9E+06	1E+07	1.12
21	Serum albumin OS	Alb	1E+07	1E+07	1.07
22	Cluster of Alpha-1-antitrypsin 1-2 OS	Serpina1b	5E+06	5E+06	1.07
23	Aspartyl aminopeptidase OS	Dnpep	1E+07	2E+07	1.17
24	Serotransferrin OS	Tf	3E+06	3E+06	1.00

25	Chymotrypsin-like elastase family member 3B OS	Cela3b	0	2E+08	
26	CUB and zona pellucida-like domain-containing protein 1 OS	Cuzd1	0	1E+07	
27	Ig kappa chain C region OS		4E+07	5E+07	1.09
28	Intestinal-type alkaline phosphatase OS	Iap	789090	895010	1.13
29	Carbonic anhydrase 6 OS	Ca6	5E+07	5E+07	1.02
30	Serpin B6 OS	Serpinb6	693210	764130	1.10
31	Mucin-13 OS	Muc13	1E+07	1E+07	1.43
32	Protein S100-A8 OS	S100a8	5E+07	5E+07	1.02
33	Anionic trypsin-2 OS	Prss2	5E+07	5E+07	1.02
34	Neutral ceramidase OS	Asah2	3E+06	3E+06	1.05
35	Annexin A2 OS	Anxa2	2E+06	2E+06	1.03
36	Cluster of Major urinary protein 18 OS	Mup18	1E+07	1E+07	1.01
37	Pregnancy zone protein OS	Pzp	326580	346630	1.06
38	Immunoglobulin J chain OS	Jchain	0	4E+07	
39	Pancreatic lipase-related protein 2 OS	Pnliprp2	0	1E+06	
40	Ig lambda-1 chain C region OS		0	4E+06	
41	Glutamyl aminopeptidase OS	Enpep	69150	72110	1.04
42	NAD-specific glutamate dehydrogenase OS	gdhB	1E+06	1E+06	1.08
43	Calcium-activated chloride channel regulator 4A OS	Clca4a	150000	160060	1.07
44	Tetraspanin-8 OS	Tspan8	4E+06	4E+06	1.09
45	Cadherin-1 OS	Cdh1	1E+06	1E+06	1.16
46	Isoform 2 of Cadherin-related family member 5 OS	Cdhr5	965075	983080	1.02
47	Epidermal growth factor receptor kinase substrate 8-like protein 3 OS	Eps8l3	669500	695050	1.04
48	Meprin A subunit alpha OS	Mep1a	0	223710	
49	Antithrombin-III OS	Serpinc1	1E+06	1E+06	1.18
50	Carboxypeptidase A1 OS	Cpa1	281790	297180	1.05
51	Choline transporter-like protein 4 OS	Slc44a4	132100	144410	1.09
52	Ferritin heavy chain OS	Fth1	1E+06	1E+06	1.07
53	Major urinary protein 20 OS	Mup20	7E+06	7E+06	1.03
54	Zinc transporter ZIP4 OS	Slc39a4	2E+06	2E+06	1.04
55	Chymotrypsinogen B OS	Ctrb1	298160	308280	1.03
56	Trefoil factor 3 OS	Tff3	1E+08	2E+08	1.08
57	Xaa-Pro aminopeptidase 2 OS	Xpnpep2	15890	16352	1.03

58	Multidrug resistance protein 1A OS	Abcb1a	31980	39167	1.22
59	Dipeptidase 1 OS	Dpep1	645100	656160	1.02
60	Isoform 2 of Meprin A subunit beta OS	Mep1b	402380	405190	1.01
61	Leukocyte elastase inhibitor A OS	Serpinb1a	348230	352720	1.01
62	Mucosal pentraxin OS	Mptx1	1E+07	1E+07	1.09
63	Ectonucleotide pyrophosphatase/phosphodiesterase family member 3 OS	Enpp3	89356	90939	1.02
64	Regenerating islet-derived protein 3-beta OS	Reg3b	562480	571450	1.02
65	Ig kappa chain V-II region 26-10 OS		2E+07	2E+07	1.01
66	Placenta-specific gene 8 protein OS	Plac8	3E+07	3E+07	1.06
67	Carboxypeptidase A2 OS	Cpa2	312540	325450	1.04
68	Prosaposin OS	Psap	312150	321730	1.03
69	Galectin-4 OS	Lgals4	911210	921140	1.01
70	Hemopexin OS	Hpx	153780	173500	1.13
71	Galectin-3-binding protein OS	Lgals3bp	63620	66274	1.04
72	Phospholipid scramblase 1 OS	Plscr1	632580	652940	1.03
73	Cluster of Ig kappa chain V-III region PC 2880/PC 1229 OS		3E+06	3E+06	1.03
74	Isoform 2 of Ig mu chain C region OS	Ighm	611700	637010	1.04
75	Hemoglobin subunit alpha OS	Hba	1E+07	2E+07	1.28
76	Murinoglobulin-1 OS	Mug1	282990	302110	1.07
77	Uromodulin OS	Umod	201000	205200	1.02
78	Hemoglobin subunit beta-1 OS	Hbb-b1	2E+06	2E+06	1.00
79	Ezrin OS	Ezr	47425	46437	0.98
80	Dipeptidyl peptidase 4 OS	Dpp4	23709	22600	0.95
81	Cluster of Ig heavy chain V region MOPC 21 (Fragment) OS		1E+06	2E+06	1.24
82	Regenerating islet-derived protein 3-gamma OS	Reg3g	2E+06	2E+06	1.08
83	Apolipoprotein A-I OS	Apoa1	1E+06	1E+06	1.08
84	Phospholipase B-like 1 OS	Plbd1	19811	18118	0.91
85	Annexin A1 OS	Anxa1	94973	97943	1.03
87	Ig kappa chain V-V region HP R16.7 OS		1E+07	9E+06	0.97
88	Sodium/potassium-transporting ATPase subunit beta-1 OS	Atp1b1	1E+06	1E+06	0.84
89	Ferritin light chain 1 OS	Ftl1	3E+06	3E+06	0.97
90	Carbonic anhydrase 4 OS	Ca4	1E+06	1E+06	1.01
91	Retinoic acid-induced protein 3 OS	Gprc5a	743200	758390	1.02

92	Isoform 2 of Phospholipase B1, membrane-associated OS	Plb1	70358	71545	1.02
93	Polyubiquitin-B OS	Ubb	3E+06	4E+06	1.24
94	Angiotensin-converting enzyme OS	Ace	43286	44489	1.03
96	Acidic mammalian chitinase OS	Chia	239990	239990	1.00
98	Alpha-2-HS-glycoprotein OS	Ahsg	139560	148240	1.06
99	Protein S100-A9 OS	S100a9	5E+06	6E+06	1.02
103	Vitamin D-binding protein OS	Gc	12155	19335	1.59
105	PDZ domain-containing protein GIPC2 OS	Gipc2	78365	80563	1.03
106	Trefoil factor 2 OS	Tff2	961700	971600	1.01
108	Ig lambda-2 chain C region OS	Iglc2	761820	798130	1.05
109	Serum amyloid P-component OS	Apcs	1E+06	1E+06	1.01
110	Protein AMBP OS	Ambp	329330	309280	0.94
111	Solute carrier family 15 member 1 OS	Slc15a1	179840	183350	1.02
113	Apolipoprotein A-II OS	Apoa2	769510	759620	0.99
114	Complement C3 OS	C3	1522.8	1325.8	0.87
115	Syncollin OS	Sycn	603400	622500	1.03

Supplementary Table 2. Fold change of miRNAs in L-Exo vs H-Exo (n=5/group) quantified by miRNA array (Qiagen).

Mature ID	L-Exo vs H-Exo	Mature ID	L-Exo vs H-Exo	Mature ID	L-Exo vs H-Exo
mmu-miR-193a-3p	139.75	mmu-miR-466g	11.46	mmu-miR-99a-5p	-79.79
mmu-miR-449a-5p	83.79	mmu-miR-9-5p	11.44	mmu-miR-455-3p	-21.76
mmu-miR-96-5p	42.95	mmu-miR-654-5p	11.43	mmu-miR-186-3p	-7.97
mmu-miR-375-3p	37.27	mmu-miR-547-3p	11.37	mmu-miR-1187	-7.91
mmu-miR-193a-3p	35.65	mmu-let-7g-3p	11.37	mmu-miR-17-3p	-7.78
mmu-miR-290a-3p	35.06	mmu-miR-383-5p	11.35	mmu-miR-130b-5p	-6.09
mmu-miR-582-3p	30.60	mmu-miR-101a-5p	10.73	mmu-miR-296-5p	-5.28
mmu-miR-500-3p	27.11	mmu-miR-470-5p	10.73	mmu-miR-1897-5p	-4.72
mmu-miR-882	24.13	mmu-miR-743a-3p	10.56	mmu-miR-1892	-4.53
mmu-miR-324-3p	24.03	mmu-miR-875-5p	10.09	mmu-miR-1194	-4.45
mmu-miR-487b-3p	22.46	mmu-miR-541-5p	10.08	mmu-let-7c-5p	-4.40
mmu-miR-874-3p	21.96	mmu-miR-133a-3p	9.90	mmu-miR-719	-4.17
mmu-miR-221-3p	21.06	mmu-miR-496a-3p	9.75	mmu-miR-467c-5p	-4.16
mmu-miR-671-3p	20.54	mmu-miR-290a-5p	9.71	mmu-miR-720	-3.95
mmu-let-7i-3p	19.43	mmu-miR-181c-5p	9.67	mmu-miR-883a-3p	-3.78
mmu-miR-10b-3p	18.50	mmu-miR-155-5p	9.54	mmu-miR-698-3p	-3.75
mmu-miR-411-5p	17.92	mmu-miR-302c-5p	9.42	mmu-miR-181a-5p	-3.60
mmu-miR-300-3p	17.20	mmu-miR-702-3p	9.27	mmu-let-7b-5p	-3.59
mmu-miR-294-5p	14.85	mmu-miR-142a-3p	9.11	mmu-let-7e-5p	-3.47
mmu-miR-743b-5p	14.51	mmu-miR-100-5p	8.74	mmu-miR-1897-3p	-3.43
mmu-miR-409-3p	13.47	mmu-miR-509-5p	8.62	mmu-miR-361-5p	-3.33
mmu-miR-15a-5p	13.24	mmu-miR-761	8.57	mmu-miR-466c-5p	-3.06
mmu-miR-345-3p	12.71	mmu-miR-210-3p	8.33	mmu-miR-146b-3p	-2.99
mmu-miR-1195	12.63	mmu-miR-665-3p	8.12	mmu-miR-196a-5p	-2.84
mmu-miR-489-3p	12.18	mmu-miR-343	8.09	mmu-miR-1895	-2.75
mmu-miR-18a-5p	12.02	mmu-miR-762	8.00	mmu-let-7d-5p	-2.74
mmu-miR-712-5p	11.94	mmu-miR-802-5p	7.77	mmu-miR-138-5p	-2.72
mmu-miR-34b-3p	11.90	mmu-miR-216a-5p	7.40	mmu-miR-23a-3p	-2.71
mmu-miR-127-5p	11.70	mmu-miR-19b-3p	7.40	mmu-miR-146b-5p	-2.70
mmu-miR-19a-3p	11.57	mmu-miR-92a-3p	7.23		

Supplementary Table 3. Lipids detected by triple quadrupole MS in CD63[+]A33[+] exosomes derived from mouse and human feces.

			Collected from mice at 6 Months after fed either RCD or HFD (nmols)		Collected from mice at 12 Months after fed either RCD or HFD (nmols)		Human (nmols)	
**Mass**	**Compound Formula**	**Compou-nd Name**	**L-Exo 6M (n=10)**	**H-Exo 6M (n=10)**	**L-Exo 12M (n=10)**	**H-Exo 12M (n=10)**	**Healthy-Exo (n=5)**	**T2D-Exo (n=7)**
494.30	C24H48O7PN	LPC(16:1)	0.01	0.00	0.01	0.62	0.03	0.00
496.30	C24H50O7PN	LPC(16:0)	0.05	0.56	0.54	56.02	7.36	0.08
518.30	C26H48O7PN	LPC(18:3)	0.01	0.13	0.00	10.66	0.12	0.03
520.30	C26H50O7PN	LPC(18:2)	0.05	0.47	0.00	106.07	2.37	0.10
522.30	C26H52O7PN	LPC(18:1)	0.01	0.27	0.01	26.23	0.65	0.05
524.40	C26H54O7PN	LPC(18:0)	0.06	1.60	0.20	13.68	0.89	0.06
542.30	C28H48O7PN	LPC(20:5)	0.01	0.00	0.01	0.00	0.02	0.00
544.30	C28H50O7PN	LPC(20:4)	0.00	0.03	0.01	0.00	0.00	0.02
546.30	C28H52O7PN	LPC(20:3)	0.00	0.00	0.00	0.00	0.00	0.00
548.40	C28H54O7PN	LPC(20:2)	0.00	0.00	0.00	0.33	0.00	0.00
550.40	C28H56O7PN	LPC(20:1)	0.00	0.08	0.00	2.30	0.08	0.00
552.40	C28H58O7PN	LPC(20:0)	0.01	0.08	0.03	3.21	0.09	0.03
568.30	C30H50O7PN	LPC(22:6)	0.01	0.00	0.00	1.86	0.07	0.03
570.30	C30H52O7PN	LPC(22:5)	0.00	0.00	0.01	0.84	0.00	0.03
Total LysoPC		Total LysoPC	0.22	3.20	0.81	221.82	0.67	0.65
676.50	C36H70O8PN	PC(28:1)	0.00	0.00	0.00	0.26	0.09	0.00
704.50	C38H74O8PN	PC(30:1)	0.00	0.02	0.09	0.32	0.02	0.00
706.50	C38H76O8PN	PC(30:0)	0.04	0.16	0.15	1.01	0.34	0.01
730.50	C40H76O8PN	PC(32:2)	0.01	0.08	0.00	0.22	0.00	0.90
732.50	C40H78O8PN	PC(32:1)	0.03	0.16	0.10	0.65	0.10	0.03
734.60	C40H80O8PN	PC(32:0)	0.09	1.34	0.32	1.72	0.40	0.42
754.50	C42H76O8PN	PC(34:4)	0.00	0.00	0.00	0.03	0.00	0.41
756.50	C42H78O8PN	PC(34:3)	0.00	0.14	0.00	1.14	0.06	0.50
758.60	C42H80O8PN	PC(34:2)	0.11	1.92	0.00	21.90	0.73	7.17
760.60	C42H82O8PN	PC(34:1)	0.12	2.26	0.10	6.39	1.28	2.17
762.60	C42H84O8PN	PC(34:0)	0.04	0.53	0.10	0.64	0.15	0.01
778.50	C44H76O8PN	PC(36:6)	0.01	0.12	0.00	0.00	0.00	0.01
780.50	C44H78O8PN	PC(36:5)	0.02	0.10	0.00	5.44	0.13	0.01
782.60	C44H80O8PN	PC(36:4)	0.06	1.60	0.00	32.76	0.15	0.05
784.60	C44H82O8PN	PC(36:3)	0.04	0.64	0.00	12.92	0.41	0.08
786.60	C44H84O8PN	PC(36:2)	0.04	0.85	0.00	7.75	0.35	0.11

788.60	C44H86O8PN	PC(36:1)	0.04	0.76	0.00	2.04	0.26	0.05
790.60	C44H88O8PN	PC(36:0)	0.03	0.40	0.00	0.52	0.15	0.01
806.60	C46H80O8PN	PC(38:6)	0.02	0.39	0.02	0.38	0.07	0.01
808.60	C46H82O8PN	PC(38:5)	0.01	0.21	0.00	0.26	0.05	0.02
810.60	C46H84O8PN	PC(38:4)	0.02	0.74	0.00	0.60	0.00	0.01
812.60	C46H86O8PN	PC(38:3)	0.02	0.29	0.00	0.80	0.07	0.02
814.60	C46H88O8PN	PC(38:2)	0.01	0.13	0.00	0.83	0.07	0.00
816.60	C46H90O8PN	PC(38:1)	0.01	0.25	0.00	0.47	0.03	0.00
818.70	C46H92O8PN	PC(38:0)	0.01	0.96	0.01	0.32	0.00	0.00
830.60	C48H80O8PN	PC(40:8)	0.00	0.00	0.00	0.29	0.05	0.00
832.60	C48H82O8PN	PC(40:7)	0.03	0.14	0.06	0.26	0.15	0.00
834.60	C48H84O8PN	PC(40:6)	0.00	0.13	0.01	0.21	0.11	0.00
836.60	C48H86O8PN	PC(40:5)	0.00	0.11	0.00	0.08	0.00	0.00
838.60	C48H88O8PN	PC(40:4)	0.00	0.00	0.00	0.06	0.00	0.00
840.60	C48H90O8PN	PC(40:3)	0.00	0.00	0.00	0.21	0.00	0.00
842.70	C48H92O8PN	PC(40:2)	0.01	0.00	0.00	0.23	0.00	0.00
852.50	C50H78O8PN	PC(42:11)	0.00	0.00	0.00	0.06	0.00	0.00
854.60	C50H80O8PN	PC(42:10)	0.00	0.00	0.00	0.10	0.00	0.00
856.60	C50H82O8PN	PC(42:9)	0.00	0.00	0.00	0.01	0.00	0.00
858.60	C50H84O8PN	PC(42:8)	0.00	0.00	0.00	0.00	0.00	0.00
860.60	C50H86O8PN	PC(42:7)	0.00	0.00	0.00	0.04	0.00	0.00
862.60	C50H88O8PN	PC(42:6)	0.00	0.00	0.00	0.14	0.00	0.00
864.60	C50H90O8PN	PC(42:5)	0.00	0.00	0.00	0.02	0.00	0.00
866.70	C50H92O8PN	PC(42:4)	0.00	0.00	0.00	0.00	0.00	0.00
868.70	C50H94O8PN	PC(42:3)	0.00	0.12	0.00	0.00	0.03	0.01
870.70	C50H96O8PN	PC(42:2)	0.00	0.00	0.00	0.04	0.00	0.00
878.60	C52H80O8PN	PC(44:12)	0.00	0.00	0.00	0.32	0.00	0.00
880.60	C52H82O8PN	PC(44:11)	0.00	0.00	0.00	0.04	0.00	0.00
882.60	C52H84O8PN	PC(44:10)	0.00	0.00	0.00	0.00	0.00	0.00
884.60	C52H86O8PN	PC(44:9)	0.00	0.00	0.00	0.00	0.00	0.00
886.60	C52H88O8PN	PC(44:8)	0.00	0.00	0.00	0.00	0.00	0.00
888.60	C52H90O8PN	PC(44:7)	0.00	0.00	0.00	0.00	0.00	0.00
890.70	C52H92O8PN	PC(44:6)	0.00	0.00	0.00	0.00	0.00	0.00
892.70	C52H94O8PN	PC(44:5)	0.00	0.00	0.01	0.00	0.00	0.00
894.70	C52H96O8PN	PC(44:4)	0.00	0.00	0.00	0.00	0.00	0.00
896.70	C52H98O8PN	PC(44:3)	0.00	0.00	0.00	0.00	0.00	0.00
898.70	C52H100O8PN	PC(44:2)	0.00	0.00	0.00	0.00	0.00	0.00
Total PC		Total PC	0.83	14.55	0.97	101.49	0.30	10.22
701.50	C39H77N2O6P	SM(16:1)	0.00	0.00	0.04	0.20	0.00	0.00

703.60	C39H79N2O6P	SM(16:0)	1.17	8.87	4.14	9.48	8.38	0.34
705.60	C39H81N2O6P	DSM(16:0)	0.39	3.08	1.22	1.55	4.51	0.25
729.60	C41H81N2O6P	SM(18:1)	0.00	0.00	0.00	0.13	0.00	0.00
731.60	C41H83N2O6P	SM(18:0)	0.03	0.44	0.08	0.55	0.62	0.06
733.60	C41H85N2O6P	DSM(18:0)	0.02	0.25	0.07	0.41	0.39	0.00
785.60	C45H89N2O6P	SM(22:1)	0.05	0.35	0.00	3.84	0.00	0.00
787.70	C45H91N2O6P	SM(22:0)	0.09	0.84	0.15	5.02	1.45	0.02
789.70	C45H93N2O6P	DSM(22:0)	0.03	0.20	0.11	1.02	0.98	0.02
813.70	C47H93N2O6P	SM(24:1)	0.14	0.28	0.45	2.42	0.57	0.04
815.70	C47H95N2O6P	SM(24:0)	0.11	0.42	0.35	2.74	1.63	0.09
817.70	C47H97N2O6P	DSM(24:0)	0.04	0.40	0.18	0.00	1.22	0.07
Total SM and DSM		Total SM and DSM	2.07	15.14	6.79	27.34	19.73	0.90
714.50	C40H76O7PN	ePC(32:3)	0.00	0.00	0.00	0.00	0.00	0.00
716.60	C40H78O7PN	ePC(32:2)	0.01	0.00	0.01	0.04	0.00	0.00
718.60	C40H80O7PN	ePC(32:1)	0.05	0.27	0.17	0.27	0.09	0.00
720.60	C40H82O7PN	ePC(32:0)	0.05	0.32	0.19	0.50	0.26	0.01
740.60	C42H78O7PN	ePC(34:4)	0.00	0.00	0.00	0.06	0.00	0.00
742.60	C42H80O7PN	ePC(34:3)	0.04	0.05	0.15	0.17	0.00	0.00
744.60	C42H82O7PN	ePC(34:2)	0.05	0.72	0.14	0.38	0.10	0.02
746.60	C42H84O7PN	ePC(34:1)	0.05	0.60	0.13	0.56	0.16	0.02
748.60	C42H86O7PN	ePC(34:0)	0.02	0.20	0.06	0.27	0.11	0.01
766.60	C44H80O7PN	ePC(36:5)	0.02	0.63	0.10	0.28	0.00	0.00
768.60	C44H82O7PN	ePC(36:4)	0.02	0.51	0.04	0.16	0.77	0.03
770.60	C44H84O7PN	ePC(36:3)	0.01	0.25	0.00	0.22	0.03	0.01
772.60	C44H86O7PN	ePC(36:2)	0.01	0.16	0.00	0.57	0.05	0.03
774.60	C44H88O7PN	ePC(36:1)	0.02	0.15	0.00	1.00	0.16	0.03
776.60	C44H90O7PN	ePC(36:0)	0.01	0.14	0.02	0.18	0.09	0.01
792.60	C46H82O7PN	ePC(38:6)	0.04	4.51	0.08	0.33	0.00	0.01
794.60	C46H84O7PN	ePC(38:5)	0.02	0.36	0.00	0.11	0.00	0.01
796.60	C46H86O7PN	ePC(38:4)	0.00	0.25	0.00	0.47	0.03	0.01
798.60	C46H88O7PN	ePC(38:3)	0.01	0.00	0.00	0.96	0.06	0.00
800.60	C46H90O7PN	ePC(38:2)	0.00	0.07	0.00	0.54	0.03	0.01
802.70	C46H92O7PN	ePC(38:1)	0.01	0.14	0.00	0.55	0.23	0.00
804.70	C46H94O7PN	ePC(38:0)	0.02	0.12	0.02	0.16	0.10	0.00
820.60	C48H86O7PN	ePC(40:6)	0.01	0.49	0.00	0.03	0.03	0.00
822.60	C48H88O7PN	ePC(40:5)	0.01	0.05	0.01	0.07	0.00	0.00
824.60	C48H90O7PN	ePC(40:4)	0.03	0.00	0.12	0.03	0.00	0.00
826.70	C48H92O7PN	ePC(40:3)	0.00	0.00	0.00	0.12	0.00	0.00
828.70	C48H94O7PN	ePC(40:2)	0.00	0.00	0.00	0.21	0.00	0.00

Total ePC		Total ePC	0.51	9.98	1.23	8.26	1.43	1.32
452.30	C21H42O7PN	LPE(16:1)	0.00	0.00	0.36	0.17	0.63	0.00
454.30	C21H44O7PN	LPE(16:0)	0.22	0.92	1.29	0.10	1.64	0.22
476.30	C23H42O7PN	LPE(18:3)	0.06	0.00	0.16	0.08	0.82	0.00
478.30	C23H44O7PN	LPE(18:2)	0.00	0.17	0.26	0.04	0.20	0.85
480.30	C23H46O7PN	LPE(18:1)	0.06	0.08	0.31	0.09	0.26	0.18
500.30	C25H42O7PN	LPE(20:5)	0.02	0.00	0.00	0.00	0.00	0.24
502.30	C25H44O7PN	LPE(20:4)	0.00	0.00	0.08	0.58	0.00	0.05
504.30	C25H46O7PN	LPE(20:3)	0.01	0.00	0.25	0.00	0.00	0.06
506.30	C25H48O7PN	LPE(20:2)	0.00	0.01	0.02	0.14	0.52	0.00
508.30	C25H50O7PN	LPE(20:1)	0.00	0.15	0.04	0.02	0.00	0.00
510.30	C25H52O7PN	LPE(20:0)	0.08	0.51	0.39	0.15	0.00	0.98
526.30	C27H44O7PN	LPE(22:6)	0.02	0.15	0.08	0.03	0.38	0.00
528.30	C27H46O7PN	LPE(22:5)	0.01	0.34	0.04	0.00	0.00	0.00
Total LysoPE		Total LysoPE	0.48	2.33	3.25	1.41	4.44	2.58
634.40	C33H64O8PN	PE(28:1)	0.00	0.00	0.00	0.00	0.18	0.00
636.50	C33H66O8PN	PE(28:0)	2.58	0.16	7.21	0.23	0.00	0.98
662.50	C35H68O8PN	PE(30:1)	0.13	0.00	0.48	0.01	0.00	0.26
664.50	C35H70O8PN	PE(30:0)	33.80	5.32	106.39	2.60	4.66	17.27
688.50	C37H70O8PN	PE(32:2)	1.23	0.00	4.18	0.11	0.00	1.66
690.50	C37H72O8PN	PE(32:1)	0.85	0.17	2.73	0.17	0.41	2.69
692.50	C37H74O8PN	PE(32:0)	2.14	0.51	6.56	0.32	0.47	1.10
712.50	C39H70O8PN	PE(34:4)	0.16	0.07	0.34	0.00	0.09	0.18
714.50	C39H72O8PN	PE(34:3)	0.58	0.00	0.53	0.00	0.74	0.84
716.50	C39H74O8PN	PE(34:2)	5.78	0.92	18.44	0.00	1.93	10.53
718.50	C39H76O8PN	PE(34:1)	2.74	0.84	8.76	0.55	1.10	7.09
720.50	C39H78O8PN	PE(34:0)	0.00	0.07	0.40	0.06	0.07	0.00
736.50	C41H70O8PN	PE(36:6)	0.04	0.01	0.04	0.00	0.00	0.08
738.50	C41H72O8PN	PE(36:5)	0.40	0.08	0.52	0.00	0.00	0.52
740.50	C41H74O8PN	PE(36:4)	0.57	0.14	1.15	0.00	0.14	0.65
742.50	C41H76O8PN	PE(36:3)	0.30	0.00	0.58	0.00	0.35	0.47
744.50	C41H78O8PN	PE(36:2)	0.30	0.13	1.05	0.40	0.34	0.40
746.60	C41H80O8PN	PE(36:1)	0.19	0.07	0.58	0.43	0.62	0.11
748.60	C41H82O8PN	PE(36:0)	0.02	0.06	0.09	0.00	0.21	0.06
764.50	C43H74O8PN	PE(38:6)	0.16	0.00	0.58	0.00	0.00	0.04
766.50	C43H76O8PN	PE(38:5)	0.13	0.02	0.45	0.04	0.00	0.04
768.50	C43H78O8PN	PE(38:4)	0.00	0.08	0.24	0.10	0.00	0.10
770.60	C43H80O8PN	PE(38:3)	0.06	0.00	0.05	0.01	0.00	0.02
772.60	C43H82O8PN	PE(38:2)	0.04	0.00	0.00	0.02	0.00	0.00

774.60	C43H84O8PN	PE(38:1)	0.01	0.00	0.00	0.01	0.09	0.00
776.60	C43H86O8PN	PE(38:0)	0.00	0.00	0.00	0.00	0.00	0.00
788.50	C45H74O8PN	PE(40:8)	0.00	0.00	0.00	0.00	0.05	0.00
790.50	C45H76O8PN	PE(40:7)	0.00	0.07	0.00	0.00	0.00	0.00
792.50	C45H78O8PN	PE(40:6)	0.00	0.00	0.00	0.01	0.00	0.00
794.60	C45H80O8PN	PE(40:5)	0.00	0.00	0.00	0.00	0.19	0.00
796.60	C45H82O8PN	PE(40:4)	0.00	0.05	0.00	0.00	0.09	0.00
798.60	C45H84O8PN	PE(40:3)	0.00	0.01	0.00	0.00	0.00	0.00
800.60	C45H86O8PN	PE(40:2)	0.00	0.00	0.00	0.00	0.00	0.00
812.50	C47H74O8PN	PE(42:10)	0.00	0.00	0.00	0.02	0.00	0.00
814.50	C47H76O8PN	PE(42:9)	0.00	0.07	0.00	0.00	0.00	0.00
816.50	C47H78O8PN	PE(42:8)	0.00	0.00	0.00	0.01	0.00	0.00
818.60	C47H80O8PN	PE(42:7)	0.00	0.13	0.00	0.00	0.00	0.00
820.60	C47H82O8PN	PE(42:6)	0.00	0.00	0.00	0.00	0.08	0.00
822.60	C47H84O8PN	PE(42:5)	0.00	0.00	0.00	0.00	0.00	0.00
824.60	C47H86O8PN	PE(42:4)	0.00	0.00	0.00	0.00	0.00	0.00
826.60	C47H88O8PN	PE(42:3)	0.00	0.00	0.00	0.00	0.00	0.00
828.60	C47H90O8PN	PE(42:2)	0.00	0.00	0.00	0.00	0.15	0.00
836.50	C49H74O8PN	PE(44:12)	0.00	0.00	0.00	0.00	0.00	0.00
838.50	C49H76O8PN	PE(44:11)	0.00	0.00	0.00	0.00	0.00	0.00
840.50	C49H78O8PN	PE(44:10)	0.00	0.00	0.00	0.00	0.00	0.00
842.60	C49H80O8PN	PE(44:9)	0.00	0.00	0.00	0.00	0.00	0.00
844.60	C49H82O8PN	PE(44:8)	0.00	0.06	0.00	0.00	0.00	0.00
846.60	C49H84O8PN	PE(44:7)	0.00	0.00	0.00	0.00	0.00	0.01
848.60	C49H86O8PN	PE(44:6)	0.00	0.02	0.00	0.00	0.00	0.00
850.60	C49H88O8PN	PE(44:5)	0.00	0.05	0.00	0.06	0.00	0.00
852.60	C49H90O8PN	PE(44:4)	0.00	0.00	0.00	0.00	0.00	0.00
854.70	C49H92O8PN	PE(44:3)	0.00	0.00	0.00	0.01	0.00	0.00
856.70	C49H94O8PN	PE(44:2)	0.00	0.00	0.00	0.00	0.00	0.00
Total PE		Total PE	52.19	9.10	161.35	5.17	18.97	45.08
659.50	C36H71O6PN2	PE-Cer(16:1)	0.02	0.00	0.05	0.01	0.00	0.05
661.50	C36H73O6PN2	PE-Cer(16:0)	0.02	0.00	0.00	0.06	0.15	0.03
687.50	C38H75O6PN2	PE-Cer(18:1)	0.53	0.07	1.35	0.01	0.05	0.33
689.50	C38H77O6PN2	PE-Cer(18:0)	0.00	0.26	0.12	0.02	0.05	0.00
773.60	C44H89O6PN2	PE-Cer(24:0)	0.00	0.00	0.00	0.00	0.00	0.00
Total PE-Cer		Total PE-Cer	0.58	0.33	1.52	0.11	0.24	0.41
672.50	C37H70O7PN	ePE(32:3)	0.49	0.00	0.78	0.06	0.00	0.20

674.50	C37H72O7PN	ePE(32:2)	0.08	0.02	0.44	0.00	0.05	0.16
676.50	C37H74O7PN	ePE(32:1)	0.45	0.00	1.88	0.00	0.00	0.97
678.50	C37H76O7PN	ePE(32:0)	21.41	3.96	65.37	1.79	5.94	14.88
698.50	C39H72O7PN	ePE(34:4)	0.04	0.20	0.12	0.02	0.00	0.04
700.50	C39H74O7PN	ePE(34:3)	0.83	0.01	2.92	0.01	0.12	0.85
702.50	C39H76O7PN	ePE(34:2)	7.56	1.04	25.15	0.56	2.14	9.04
704.60	C39H78O7PN	ePE(34:1)	2.64	1.22	8.63	0.34	1.23	5.57
706.60	C39H80O7PN	ePE(34:0)	0.26	0.00	0.89	0.04	0.00	0.16
724.50	C41H74O7PN	ePE(36:5)	0.45	0.00	1.45	0.06	0.12	0.31
726.50	C41H76O7PN	ePE(36:4)	0.63	0.00	1.87	0.02	0.00	0.50
728.60	C41H78O7PN	ePE(36:3)	0.28	0.19	1.36	0.03	0.28	0.18
730.60	C41H80O7PN	ePE(36:2)	0.39	0.13	1.34	0.12	0.00	0.53
732.60	C41H82O7PN	ePE(36:1)	0.22	0.00	0.72	0.10	0.08	0.38
734.60	C41H84O7PN	ePE(36:0)	0.01	0.03	0.08	0.00	0.00	0.01
750.50	C43H76O7PN	ePE(38:6)	0.26	0.00	1.03	0.01	0.00	0.00
752.60	C43H78O7PN	ePE(38:5)	0.21	0.00	0.74	0.03	0.05	0.06
754.60	C43H80O7PN	ePE(38:4)	0.09	0.00	0.25	0.00	0.00	0.14
756.60	C43H82O7PN	ePE(38:3)	0.00	0.09	0.07	0.00	0.00	0.00
758.60	C43H84O7PN	ePE(38:2)	0.00	0.07	0.00	0.02	0.00	0.07
760.60	C43H86O7PN	ePE(38:1)	0.01	0.00	0.07	0.00	0.00	0.07
762.60	C43H88O7PN	ePE(38:0)	0.04	0.03	0.00	0.00	0.00	0.04
778.60	C45H80O7PN	ePE(40:6)	0.00	0.00	0.04	0.01	0.02	0.00
780.60	C45H82O7PN	ePE(40:5)	0.00	0.11	0.05	0.01	0.00	0.02
782.60	C45H84O7PN	ePE(40:4)	0.00	0.00	0.00	0.03	0.00	0.01
784.60	C45H86O7PN	ePE(40:3)	0.00	0.00	0.00	0.00	0.00	0.00
786.60	C45H88O7PN	ePE(40:2)	0.00	0.00	0.00	0.00	0.00	0.00
Total ePE		Total ePE	36.34	7.11	115.24	3.27	10.02	24.17
848.50	C43H75O13P	PI(34:4)	0.00	0.00	0.02	0.00	0.00	0.14
850.50	C43H77O13P	PI(34:3)	0.01	0.00	0.00	0.00	0.38	0.23
852.50	C43H79O13P	PI(34:2)	0.14	0.32	0.00	0.00	0.37	0.36
854.50	C43H81O13P	PI(34:1)	0.04	0.30	0.09	0.30	0.00	0.20
872.50	C45H75O13P	PI(36:6)	0.02	0.32	0.00	0.00	0.01	0.32
874.50	C45H77O13P	PI(36:5)	0.01	0.12	0.00	0.00	0.11	0.00
876.50	C45H79O13P	PI(36:4)	0.04	0.63	0.06	0.12	0.30	0.15
878.50	C45H81O13P	PI(36:3)	0.02	0.00	0.06	0.15	1.12	0.08
880.60	C45H83O13P	PI(36:2)	0.09	0.45	0.13	0.62	0.56	0.00
882.60	C45H85O13P	PI(36:1)	0.01	0.16	0.01	0.52	0.06	0.00
900.50	C47H79O13P	PI(38:6)	0.04	0.16	0.04	0.00	0.09	0.06
902.50	C47H81O13P	PI(38:5)	0.03	0.03	0.04	0.07	0.13	0.04

904.60	C47H83O13P	PI(38:4)	0.04	0.54	0.13	0.59	0.00	0.10
906.60	C47H85O13P	PI(38:3)	0.04	0.30	0.15	0.27	0.75	0.00
908.60	C47H87O13P	PI(38:2)	0.00	0.00	0.00	0.02	0.00	0.09
910.60	C47H89O13P	PI(38:1)	0.01	0.12	0.00	0.07	0.00	0.03
912.60	C47H91O13P	PI(38:0)	0.02	0.00	0.00	0.00	0.03	0.00
924.50	C49H79O13P	PI(40:8)	0.01	0.19	0.04	0.02	0.05	0.00
926.50	C49H81O13P	PI(40:7)	0.00	0.52	0.01	0.07	0.39	0.08
928.60	C49H83O13P	PI(40:6)	0.01	0.30	0.06	0.00	0.03	0.06
930.60	C49H85O13P	PI(40:5)	0.00	0.32	0.00	0.00	0.00	0.11
932.60	C49H87O13P	PI(40:4)	0.02	0.17	0.00	0.07	0.05	0.06
934.60	C49H89O13P	PI(40:3)	0.00	0.05	0.01	0.00	0.31	0.08
936.60	C49H91O13P	PI(40:2)	0.00	0.17	0.05	0.09	0.06	0.08
938.60	C49H93O13P	PI(40:1)	0.01	0.00	0.01	0.04	0.35	0.07
940.70	C49H95O13P	PI(40:0)	0.04	0.09	0.01	0.05	0.21	0.08
948.50	C51H79O13P	PI(42:10)	0.01	0.00	0.00	0.03	0.01	0.09
950.50	C51H81O13P	PI(42:9)	0.00	0.11	0.00	0.00	0.04	0.01
952.60	C51H83O13P	PI(42:8)	0.01	0.63	0.01	0.05	0.21	0.14
954.60	C51H85O13P	PI(42:7)	0.01	0.40	0.02	0.00	0.00	0.04
956.60	C51H87O13P	PI(42:6)	0.00	0.11	0.01	0.00	0.21	0.14
958.60	C51H89O13P	PI(42:5)	0.00	0.00	0.00	0.03	0.23	0.05
960.60	C51H91O13P	PI(42:4)	0.01	0.07	0.00	0.01	0.00	0.16
962.60	C51H93O13P	PI(42:3)	0.05	0.03	0.03	0.06	0.00	0.00
964.70	C51H95O13P	PI(42:2)	0.00	0.05	0.00	0.01	0.00	0.12
972.50	C53H79O13P	PI(44:12)	0.00	0.49	0.02	0.15	0.09	0.01
974.50	C53H81O13P	PI(44:11)	0.01	0.00	0.00	0.00	0.08	0.03
976.60	C53H83O13P	PI(44:10)	0.01	0.11	0.01	0.01	0.09	0.07
978.60	C53H85O13P	PI(44:9)	0.01	0.03	0.00	0.01	0.01	0.07
980.60	C53H87O13P	PI(44:8)	0.00	0.03	0.00	0.06	0.00	0.23
982.60	C53H89O13P	PI(44:7)	0.04	0.00	0.01	0.00	0.01	0.00
984.60	C53H91O13P	PI(44:6)	0.06	0.24	0.07	0.02	0.03	0.00
986.60	C53H93O13P	PI(44:5)	0.00	0.00	0.02	0.03	0.06	0.10
988.70	C53H95O13P	PI(44:4)	0.02	0.36	0.00	0.02	0.17	0.38
990.70	C53H97O13P	PI(44:3)	0.00	0.04	0.01	0.07	0.26	0.00
992.70	C53H99O13P	PI(44:2)	0.00	0.01	0.02	0.01	0.00	0.20
Total PI		Total PI	0.92	7.96	1.16	3.64	6.89	4.26
734.50	C38H72O10PN	PS(32:1)	0.00	0.00	0.23	0.00	0.00	0.05
736.50	C38H74O10PN	PS(32:0)	0.23	1.00	0.57	0.19	0.81	0.19
756.50	C40H70O10PN	PS(34:4)	0.04	0.00	0.02	0.00	0.02	0.04
758.50	C40H72O10PN	PS(34:3)	0.02	0.00	0.04	0.00	0.00	0.00

760.50	C40H74O10PN	PS(34:2)	0.50	0.64	1.38	0.36	0.37	0.26
762.50	C40H76O10PN	PS(34:1)	0.12	0.00	0.67	0.18	0.93	0.32
764.50	C40H78O10PN	PS(34:0)	0.00	0.07	0.00	0.00	0.00	0.03
780.50	C42H70O10PN	PS(36:6)	0.00	0.07	0.03	0.00	0.06	0.07
782.50	C42H72O10PN	PS(36:5)	0.03	0.00	0.02	0.06	0.04	0.10
784.50	C42H74O10PN	PS(36:4)	0.01	0.02	0.07	0.04	0.09	0.00
786.50	C42H76O10PN	PS(36:3)	0.03	0.00	0.09	0.03	0.38	0.01
788.50	C42H78O10PN	PS(36:2)	0.17	1.16	0.33	0.19	0.80	0.36
790.60	C42H80O10PN	PS(36:1)	1.15	3.32	1.09	0.30	8.44	3.94
792.60	C42H82O10PN	PS(36:0)	0.02	0.64	0.00	0.01	0.89	0.06
806.50	C44H72O10PN	PS(38:7)	0.00	0.00	0.00	0.03	0.00	0.02
808.50	C44H74O10PN	PS(38:6)	0.00	0.13	0.00	0.06	0.06	0.00
810.50	C44H76O10PN	PS(38:5)	0.01	0.00	0.02	0.03	0.00	0.04
812.50	C44H78O10PN	PS(38:4)	0.03	0.00	0.00	0.00	0.02	0.00
814.60	C44H80O10PN	PS(38:3)	0.17	0.39	0.44	0.05	0.54	0.08
816.60	C44H82O10PN	PS(38:2)	0.05	0.29	0.08	0.00	0.09	0.03
818.60	C44H84O10PN	PS(38:1)	0.07	0.60	0.12	0.00	0.00	0.17
820.60	C44H86O10PN	PS(38:0)	0.04	0.00	0.00	0.02	0.15	0.16
832.50	C46H74O10PN	PS(40:8)	0.00	0.00	0.03	0.07	0.10	0.00
834.50	C46H76O10PN	PS(40:7)	0.04	0.00	0.02	0.03	0.00	0.02
836.50	C46H78O10PN	PS(40:6)	0.00	0.00	0.13	0.02	0.00	0.00
838.60	C46H80O10PN	PS(40:5)	0.01	0.00	0.01	0.02	0.15	0.00
840.60	C46H82O10PN	PS(40:4)	0.00	0.00	0.00	0.00	0.00	0.05
842.60	C46H84O10PN	PS(40:3)	0.01	0.09	0.01	0.00	0.00	0.00
844.60	C46H86O10PN	PS(40:2)	0.06	0.17	0.00	0.04	0.39	0.02
846.60	C46H88O10PN	PS(40:1)	0.12	0.14	0.27	0.08	0.08	0.08
854.50	C48H72O10PN	PS(42:11)	0.00	0.06	0.00	0.00	0.00	0.05
856.50	C48H74O10PN	PS(42:10)	0.00	0.00	0.00	0.00	0.00	0.04
858.50	C48H76O10PN	PS(42:9)	0.00	0.21	0.05	0.00	0.00	0.02
860.50	C48H78O10PN	PS(42:8)	0.00	0.00	0.03	0.00	0.01	0.07
862.60	C48H80O10PN	PS(42:7)	0.01	0.01	0.05	0.00	0.00	0.00
864.60	C48H82O10PN	PS(42:6)	0.01	0.00	0.00	0.00	0.11	0.00
866.60	C48H84O10PN	PS(42:5)	0.00	0.01	0.00	0.00	0.17	0.05
880.50	C50H74O10PN	PS(44:12)	0.01	0.03	0.00	0.00	0.00	0.01
882.50	C50H76O10PN	PS(44:11)	0.01	0.03	0.02	0.00	0.29	0.00
884.50	C50H78O10PN	PS(44:10)	0.00	0.00	0.01	0.00	0.00	0.02
886.60	C50H80O10PN	PS(44:9)	0.00	0.12	0.03	0.00	0.00	0.00
888.60	C50H82O10PN	PS(44:8)	0.00	0.00	0.02	0.01	0.08	0.00
890.60	C50H84O10PN	PS(44:7)	0.00	0.04	0.00	0.02	0.01	0.00

892.60	C50H86O10PN	PS(44:6)	0.02	0.06	0.02	0.00	0.00	0.05
894.60	C50H88O10PN	PS(44:5)	0.00	0.00	0.00	0.00	0.10	0.00
896.60	C50H90O10PN	PS(44:4)	0.00	0.00	0.02	0.00	0.00	0.01
898.60	C50H92O10PN	PS(44:3)	0.00	0.00	0.00	0.00	0.06	0.01
900.70	C50H94O10PN	PS(44:2)	0.00	0.27	0.01	0.03	0.03	0.03
Total PS		Total PS	3.01	9.58	5.94	1.88	15.27	6.45
746.50	C40H76O9PN	ePS(34:2)	0.51	1.91	1.46	0.46	0.82	0.79
748.50	C40H78O9PN	ePS(34:1)	0.23	0.34	0.50	0.04	0.04	0.17
772.50	C42H78O9PN	ePS(36:3)	0.04	0.06	0.03	0.04	0.00	0.01
774.60	C42H80O9PN	ePS(36:2)	0.02	0.00	0.12	0.04	0.00	0.02
776.60	C42H82O9PN	ePS(36:1)	0.07	0.23	0.06	0.04	0.45	0.34
778.60	C42H84O9PN	ePS(36:0)	0.02	0.00	0.00	0.01	0.06	0.01
794.50	C44H76O9PN	ePS(38:6)	0.03	0.00	0.05	0.01	0.01	0.04
796.50	C44H78O9PN	ePS(38:5)	0.00	0.04	0.06	0.00	0.00	0.00
798.60	C44H80O9PN	ePS(38:4)	0.01	0.00	0.00	0.00	0.00	0.07
800.60	C44H82O9PN	ePS(38:3)	0.00	0.00	0.00	0.00	0.00	0.00
802.60	C44H84O9PN	ePS(38:2)	0.00	0.00	0.00	0.00	0.00	0.00
804.60	C44H86O9PN	ePS(38:1)	0.02	0.24	0.01	0.04	0.00	0.05
824.60	C46H82O9PN	ePS(40:5)	0.00	0.00	0.00	0.00	0.00	0.00
826.60	C46H84O9PN	ePS(40:4)	0.00	0.00	0.00	0.00	0.00	0.06
828.60	C46H86O9PN	ePS(40:3)	0.00	0.00	0.01	0.00	0.52	0.08
830.60	C46H88O9PN	ePS(40:2)	0.01	0.32	0.00	0.00	0.00	0.00
Total ePS		Total ePS	0.97	3.16	2.30	0.67	1.91	1.64
658.40	C35H61O8P	PA(32:4)	0.00	0.03	0.01	0.02	0.01	0.00
660.40	C35H63O8P	PA(32:3)	0.00	0.00	0.00	0.01	0.11	0.03
662.40	C35H65O8P	PA(32:2)	0.04	0.07	0.10	0.00	0.00	0.04
664.50	C35H67O8P	PA(32:1)	0.01	0.28	0.08	0.19	0.03	0.06
666.50	C35H69O8P	PA(32:0)	0.10	0.32	0.34	0.37	0.07	0.10
686.40	C37H65O8P	PA(34:4)	0.00	0.10	0.00	0.00	0.07	0.05
688.50	C37H67O8P	PA(34:3)	0.01	0.02	0.07	0.09	0.11	0.00
690.50	C37H69O8P	PA(34:2)	0.20	1.14	0.74	0.55	0.74	0.15
692.50	C37H71O8P	PA(34:1)	0.09	0.94	0.38	0.89	0.26	0.19
710.40	C39H65O8P	PA(36:6)	0.00	0.02	0.00	0.00	0.02	0.03
712.50	C39H67O8P	PA(36:5)	0.04	0.00	0.06	0.09	0.03	0.08
714.50	C39H69O8P	PA(36:4)	0.17	0.31	0.22	0.42	0.47	0.10
716.50	C39H71O8P	PA(36:3)	0.06	0.40	0.21	0.31	0.12	0.04
718.50	C39H73O8P	PA(36:2)	0.05	1.82	0.00	0.41	0.29	0.12
738.50	C41H69O8P	PA(38:6)	0.01	0.06	0.02	0.00	0.06	0.02
740.50	C41H71O8P	PA(38:5)	0.00	0.33	0.02	0.04	0.00	0.02

742.50	C41H73O8P	PA(38:4)	0.00	0.62	0.01	0.24	0.07	0.00
744.50	C41H75O8P	PA(38:3)	0.01	0.12	0.03	0.44	0.12	0.02
746.50	C41H77O8P	PA(38:2)	0.00	0.00	0.00	0.12	0.04	0.02
764.50	C43H71O8P	PA(40:7)	0.00	0.00	0.02	0.01	0.00	0.00
766.50	C43H73O8P	PA(40:6)	0.01	0.15	0.02	0.03	0.00	0.00
768.50	C43H75O8P	PA(40:5)	0.01	0.02	0.01	0.04	0.05	0.02
Total PA		Total PA	0.82	6.76	2.36	4.27	2.68	1.07
710.50	C36H69O10P	PG(30:1)	0.04	0.00	0.07	0.08	0.05	0.12
712.50	C36H71O10P	PG(30:0)	0.05	0.40	0.56	2.25	0.84	0.17
736.50	C38H71O10P	PG(32:2)	0.03	0.00	0.05	0.10	0.08	0.00
738.50	C38H73O10P	PG(32:1)	0.02	0.56	0.00	0.34	0.69	0.10
740.50	C38H75O10P	PG(32:0)	0.20	1.39	0.60	2.99	0.85	0.40
760.50	C40H71O10P	PG(34:4)	0.00	0.08	0.00	0.00	0.00	0.08
762.50	C40H73O10P	PG(34:3)	0.01	0.00	0.00	0.00	0.10	0.01
764.50	C40H75O10P	PG(34:2)	0.07	0.45	0.00	0.00	0.52	0.09
766.50	C40H77O10P	PG(34:1)	0.40	3.77	1.35	1.77	1.40	0.40
768.50	C40H79O10P	PG(34:0)	0.12	0.90	0.27	1.75	0.93	0.29
788.50	C42H75O10P	PG(36:4)	0.01	0.00	0.00	0.11	0.55	0.08
790.50	C42H77O10P	PG(36:3)	0.00	0.16	0.06	0.16	0.05	0.04
792.50	C42H79O10P	PG(36:2)	0.07	1.07	0.46	0.76	1.49	0.08
794.60	C42H81O10P	PG(36:1)	0.04	2.01	0.27	0.80	0.10	0.07
812.50	C44H75O10P	PG(38:6)	0.00	0.02	0.00	0.00	0.02	0.00
814.50	C44H77O10P	PG(38:5)	0.00	0.00	0.00	0.00	0.00	0.00
Total PG		Total PG	1.07	10.81	3.67	11.12	7.67	1.93
30:1	C33H66O5N	14:0/16:1	0.07	0.26	0.00	0.00	0.51	2.45
		16:0/14:1	0.11	0.31	4.88	15.06	2.55	2.77
30:0	C33H68O5N	14:0/16:0			14.84	82.32		
32:1	C35H70O5N	16:0/16:1	0.48	2.06	0.00	10.91	6.55	4.28
32:0	C35H72O5N	16:0/16:0	0.36	1.18	0.00	307.31	2.02	2.62
34:4	C37H68O5N	18:3/16:1	0.81	1.57	0.18	15.88	4.38	10.03
		14:0/20:4	0.05	1.36	1.22	3.97	0.34	0.95
		14:1/20:3	0.04	0.09	0.79	7.42	0.82	0.27
34:3	C37H70O5N	18:3/16:0	0.22	2.83	7.00	49.67	0.31	0.87
		18:2/16:1	0.10	0.67	0.00	15.76	1.48	0.97
		14:0/20:3	0.31	1.67	0.22	1.85	0.92	0.95
34:2	C37H72O5N	18:2/16:0	0.03	0.00	66.53	354.97	0.17	0.41
		18:1/16:1	4.93	10.76	0.00	42.51	18.98	5.30
34:1	C37H74O5N	18:1/16:0	0.86	0.46	28.95	663.49	2.15	2.70

		18:0/16:1	7.07	7.91	0.00	24.59	10.68	9.18
36:6	C39H68O5N	18:3/18:3	0.41	0.95	0.00	0.00	2.20	5.37
		16:1/20:5	0.04	0.00	0.54	10.94	0.09	0.73
36:5	C39H70O5N	18:3/18:2	0.15	0.86	0.98	32.16	0.70	1.36
		16:0/20:5	0.21	0.55	1.05	0.00	0.04	1.04
		16:1/20:4	0.70	4.52	0.00	6.90	0.71	0.29
36:4	C39H72O5N	18:3/18:1	0.03	0.34	1.57	81.19	0.39	0.34
		18:2/18:2	0.81	1.84	1.17	58.75	0.78	0.17
		16:0/20:4	3.16	4.32	19.85	35.57	0.86	1.02
		16:1/20:3	0.08	0.28	0.00	6.63	4.14	1.24
36:3	C39H74O5N	18:3/18:0	0.11	0.30	1.76	44.65	0.00	0.00
		18:2/18:1	0.21	0.24	2.92	196.85	0.27	0.49
		16:0/20:3	20.51	8.71	2.44	20.47	2.39	1.17
		16:1/20:2	0.14	0.06	0.04	7.11	0.76	0.12
36:2	C39H76O5N	18:2/18:0	0.29	0.07	10.21	92.47	0.55	1.75
		18:1/18:1	1.74	4.41	1.12	180.29	2.13	4.76
		16:0/20:2	40.10	10.81	1.04	22.29	4.17	2.19
36:1	C39H78O5N	18:1/18:0	0.02	0.36	0.00	137.62	0.43	1.38
38:7	C41H70O5N	18:2/20:5	4.89	2.59	0.67	2.31	3.64	7.56
		18:3/20:4	2.74	3.14	0.00	2.97	0.22	0.02
38:6	C41H72O5N	18:1/20:5	0.02	0.00	0.07	3.46	0.00	0.00
		18:2/20:4	3.40	4.29	0.00	6.20	0.52	0.80
		16:0/22:6	0.08	1.50	3.83	7.02	0.04	0.32
		16:1/22:5	0.00	0.30	0.00	4.17	0.83	1.31
38:5	C41H74O5N	18:0/20:5	0.05	0.00	0.23	4.95	0.20	0.41
		18:1/20:4	1.45	6.63	1.35	13.83	0.32	0.68
		18:2/20:3	0.14	0.33	0.39	0.66	0.52	0.29
		18:3/20:2	0.04	0.04	0.00	0.00	0.04	0.17
		16:0/22:5	0.04	0.07	27.10	4.91	0.00	0.58
		16:1/22:4	0.00	0.15	0.00	3.48	6.69	0.07
38:4	C41H76O5N	18:0/20:4	0.09	0.50	6.02	53.29	0.34	0.95
		18:1/20:3	0.10	0.23	0.54	6.39	0.71	1.46
		16:0/22:4	0.21	0.54	22.35	18.15	0.10	0.24
38:3	C41H78O5N	18:0/20:3	0.14	0.68	5.07	27.49	5.81	4.03
		18:1/20:2	0.10	0.22	0.05	14.28	0.98	0.87
40:7	C43H74O5N	18:1/22:6	0.11	0.39	0.00	0.00	0.00	0.63
		18:2/22:5	0.23	0.28	0.00	0.00	0.33	2.31
		18:3/22:4	0.21	0.46	0.00	0.00	1.41	2.99
		20:2/20:5	0.00	0.09	0.00	0.00	0.00	0.00

		20:3/20:4	0.05	0.18	0.00	0.00	0.00	0.00
40:6	C43H76O5N	18:0/22:6	0.02	0.36	0.00	0.00	0.00	0.19
		18:1/22:5	0.23	0.50	0.00	0.00	0.72	3.96
		18:2/22:4	0.23	0.38	0.00	0.00	1.28	0.85
		20:2/20:4	0.67	3.57	0.00	0.00	0.57	0.70
		20:3/20:3	0.00	0.00	0.00	0.00	0.00	0.00
40:5	C43H78O5N	18:0/22:5	0.00	0.00	0.00	0.00	0.00	0.00
		18:1/22:4	0.00	0.29	0.00	0.00	0.99	0.10
		20:2/20:3	0.62	2.53	0.00	0.00	1.17	1.09
		Total DAG	0.00	0.00	236.98	270.32	0.12	0.22
764.67	C47H90O6N	44:2	100.00	100.00	0.13	0.01	100.00	100.00
766.69	C47H92O6N	44:1	0.00	0.01	0.00	0.01	0.83	0.01
768.71	C47H94O6N	44:0	0.00	0.00	0.05	0.00	0.03	0.00
776.71	C49H94O5N	e46:3	0.00	0.00	0.00	0.00	0.51	0.00
778.73	C49H96O5N	e46:2	0.00	0.00	0.07	0.01	0.00	0.00
780.74	C49H98O5N	e46:1	0.00	0.01	0.00	0.00	0.44	0.01
782.76	C49H100O5N	e46:0	0.00	0.00	0.13	0.00	1.71	0.00
790.69	C49H92O6N	46:3	0.00	0.00	0.00	0.00	1.02	0.00
792.71	C49H94O6N	46:2	0.00	0.00	0.01	0.00	0.00	0.00
794.72	C49H96O6N	46:1	0.00	0.00	0.00	0.02	0.08	0.00
796.74	C49H98O6N	46:0	0.00	0.00	0.06	0.00	0.04	0.01
802.73	C51H96O5N	e48:4	0.00	0.00	0.00	0.00	0.93	0.00
804.74	C51H98O5N	e48:3	0.00	0.00	0.00	0.00	0.00	0.00
806.76	C51H100O5N	e48:2	0.00	0.00	0.00	0.00	0.06	0.00
808.77	C51H102O5N	e48:1	0.00	0.00	0.00	0.00	0.03	0.00
810.79	C51H104O5N	e48:0	0.00	0.00	0.01	0.00	0.00	0.00
816.71	C51H94O6N	48:4	0.00	0.00	0.00	0.00	0.13	0.00
818.72	C51H96O6N	48:3	0.00	0.00	0.00	0.00	0.00	0.00
820.74	C51H98O6N	48:2	0.00	0.00	0.00	0.01	0.02	0.00
822.75	C51H100O6N	48:1	0.00	0.00	0.00	0.01	0.02	0.00
824.77	C51H102O6N	48:0	0.00	0.00	0.00	0.00	0.01	0.00
830.76	C53H100O5N	e50:4	0.00	0.00	0.00	0.00	0.00	0.00
832.77	C53H102O5N	e50:3	0.00	0.00	0.00	0.00	0.00	0.00
834.79	C53H104O5N	e50:2	0.00	0.00	0.00	0.00	0.00	0.01
836.80	C53H106O5N	e50:1	0.00	0.00	0.00	0.00	0.01	0.00
838.82	C53H108O5N	e50:0	0.00	0.00	0.00	0.00	0.00	0.00
844.74	C53H98O6N	50:4	0.00	0.00	0.00	0.00	0.00	0.00
846.75	C53H100O6N	50:3	0.00	0.00	0.00	0.00	0.00	0.00
848.77	C53H102O6N	50:2	0.00	0.00	0.00	0.01	0.00	0.00

850.78	C53H104O6N	50:1	0.00	0.00	0.00	0.00	0.00	0.00
852.80	C53H106O6N	50:0	0.00	0.00	0.00	0.00	0.01	0.00
856.77	C55H102O5N	e52:5	0.00	0.00	0.00	0.00	0.00	0.00
858.79	C55H104O5N	e52:4	0.00	0.00	0.00	0.00	0.00	0.00
860.80	C55H106O5N	e52:3	0.00	0.00	0.00	0.01	0.00	0.00
862.82	C55H108O5N	e52:2	0.00	0.00	0.00	0.00	0.00	0.00
864.84	C55H110O5N	e52:1	0.00	0.00	0.00	0.00	0.00	0.00
866.85	C55H112O5N	e52:0	0.00	0.00	0.00	0.00	0.06	0.00
868.74	C55H98O6N	52:6	0.00	0.00	0.00	0.00	0.01	0.00
870.75	C55H100O6N	52:5	0.00	0.00	0.00	0.00	0.01	0.00
872.77	C55H102O6N	52:4	0.00	0.00	0.00	0.00	0.00	0.00
874.78	C55H104O6N	52:3	0.00	0.00	0.00	0.00	0.00	0.00
876.80	C55H106O6N	52:2	0.00	0.00	0.00	0.00	0.01	0.00
878.81	C55H108O6N	52:1	0.00	0.00	0.00	0.00	0.00	0.00
880.83	C55H110O6N	52:0/e54:7	0.00	0.00	0.00	0.00	0.00	0.00
882.79	C57H104O5N	e54:6	0.00	0.00	0.00	0.00	0.01	0.00
884.80	C57H106O5N	e54:5	0.00	0.01	0.00	0.00	0.00	0.00
886.82	C57H108O5N	e54:4	0.00	0.00	0.00	0.00	0.00	0.00
888.84	C57H110O5N	e54:3	0.00	0.00	0.00	0.00	0.00	0.00
890.85	C57H112O5N	e54:2	0.00	0.00	0.00	0.00	0.00	0.00
892.87	C57H114O5N	e54:1	0.00	0.00	0.00	0.00	0.00	0.00
894.75	C57H100O6N	54:7/e54:0	0.00	0.00	0.00	0.00	0.00	0.00
896.77	C57H102O6N	54:6	0.00	0.00	0.00	0.00	0.00	0.00
898.78	C57H104O6N	54:5	0.00	0.00	0.00	0.00	0.00	0.00
900.80	C57H106O6N	54:4	0.00	0.00	0.00	0.00	0.00	0.00
902.81	C57H108O6N	54:3	0.00	0.00	0.00	0.00	0.00	0.00
904.83	C57H110O6N	54:2	0.00	0.00	0.00	0.00	0.00	0.00
906.85	C57H112O6N	54:1	0.00	0.00	0.00	0.00	0.00	0.00
908.86	C57H114O6N	54:0/e56:7	0.00	0.00	0.00	0.00	0.00	0.00
910.82	C59H108O5N	e56:6	0.00	0.00	0.00	0.00	0.00	0.00
912.84	C59H110O5N	e56:5	0.00	0.00	0.00	0.00	0.01	0.00
914.85	C59H112O5N	e56:4	0.00	0.00	0.00	0.00	0.00	0.00
916.87	C59H114O5N	e56:3	0.00	0.00	0.00	0.00	0.00	0.00
918.88	C59H116O5N	e56:2	0.00	0.00	0.00	0.00	0.00	0.00
920.90	C59H118O5N	e56:1	0.00	0.00	0.00	0.00	0.00	0.00
922.78	C59H104O6N	56:7/e56:0	0.00	0.00	0.00	0.00	0.01	0.00
924.80	C59H106O6N	56:6	0.00	0.00	0.00	0.00	0.00	0.00
926.81	C59H108O6N	56:5	0.00	0.00	0.00	0.00	0.00	0.00
928.83	C59H110O6N	56:4	0.00	0.00	0.00	0.00	0.00	0.00

930.85	C59H112O6N	56:3	0.00	0.00	0.00	0.00	0.03	0.00
932.86	C59H114O6N	56:2	0.00	0.00	0.00	0.00	0.00	0.00
934.88	C59H116O6N	56:1	0.00	0.00	0.00	0.00	0.00	0.00
936.84	C61H110O5N	e58:7/56:0	0.00	0.00	0.00	0.00	0.00	0.00
938.85	C61H112O5N	e58:6	0.00	0.00	0.00	0.00	0.00	0.00
940.87	C61H114O5N	e58:5	0.00	0.00	0.00	0.00	0.00	0.00
942.88	C61H116O5N	e58:4	0.00	0.00	0.00	0.00	0.00	0.00
944.90	C61H118O5N	e58:3	0.00	0.00	0.00	0.00	0.01	0.00
946.91	C61H120O5N	e58:2	0.00	0.00	0.00	0.00	0.00	0.00
948.93	C61H122O5N	e58:1/58:8	0.00	0.00	0.00	0.00	0.01	0.00
950.81	C61H108O6N	58:7/e58:0	0.00	0.00	0.00	0.00	0.00	0.00
952.83	C61H110O6N	58:6	0.00	0.00	0.00	0.00	0.01	0.00
954.85	C61H112O6N	58:5	0.00	0.00	0.00	0.00	0.00	0.00
956.86	C61H114O6N	58:4	0.00	0.00	0.00	0.00	0.00	0.00
958.88	C61H116O6N	58:3	0.00	0.00	0.00	0.00	0.00	0.00
960.89	C61H118O6N	58:2	0.00	0.00	0.00	0.00	0.00	0.00
962.91	C61H120O6N	58:1	0.00	0.00	0.00	0.00	0.01	0.00
964.87	C63H114O5N	e60:7/58:0	0.00	0.00	0.00	0.00	0.01	0.00
966.88	C63H116O5N	e60:6	0.00	0.00	0.00	0.00	0.00	0.00
968.90	C63H118O5N	e60:5	0.00	0.00	0.00	0.00	0.00	0.00
970.91	C63H120O5N	e60:4	0.00	0.00	0.00	0.00	0.00	0.00
972.93	C63H122O5N	e60:3	0.00	0.00	0.00	0.00	0.00	0.00
974.94	C63H124O5N	e60:2	0.00	0.00	0.00	0.00	0.00	0.00
976.83	C63H110O6N	60:8/e60:1	0.00	0.00	0.00	0.00	0.00	0.00
978.85	C63H112O6N	60:7/e60:0	0.00	0.00	0.00	0.00	0.01	0.00
980.86	C63H114O6N	60:6	0.00	0.00	0.00	0.00	0.00	0.00
982.88	C63H116O6N	60:5	0.00	0.00	0.00	0.00	0.00	0.00
984.89	C63H118O6N	60:4	0.00	0.00	0.00	0.00	0.00	0.00
986.91	C63H120O6N	60:3	0.00	0.00	0.00	0.00	0.00	0.00
988.92	C63H122O6N	60:2	0.00	0.00	0.00	0.00	0.03	0.00
990.94	C63H124O6N	60:1	0.00	0.00	0.00	0.00	0.00	0.00
Total NL 243 14:1 acyl containing		Total NL 243 14:1 acyl containing	0.00	0.00	0.52	0.15	0.01	0.00
764.67	C47H90O6N	44:2	0.06	0.11	0.00	0.00	6.13	0.10
766.69	C47H92O6N	44:1	0.00	0.00	0.01	0.04	0.00	0.00
768.71	C47H94O6N	44:0	0.00	0.00	0.01	0.27	0.08	0.00
776.71	C49H94O5N	e46:3	0.01	0.02	0.00	0.00	0.25	0.03
778.73	C49H96O5N	e46:2	0.00	0.00	0.00	0.00	0.00	0.00

780.74	C49H98O5N	e46:1	0.00	0.00	0.00	0.00	0.02	0.00
782.76	C49H100O5N	e46:0	0.01	0.01	0.01	0.04	0.21	0.02
790.69	C49H92O6N	46:3	0.00	0.00	0.00	0.00	0.12	0.01
792.71	C49H94O6N	46:2	0.00	0.00	0.00	0.01	0.00	0.00
794.72	C49H96O6N	46:1	0.00	0.00	0.00	0.07	0.04	0.00
796.74	C49H98O6N	46:0	0.01	0.01	0.02	0.38	0.05	0.01
802.73	C51H96O5N	e48:4	0.02	0.08	0.00	0.00	0.30	0.11
804.74	C51H98O5N	e48:3	0.00	0.00	0.00	0.00	0.01	0.00
806.76	C51H100O5N	e48:2	0.00	0.00	0.00	0.00	0.00	0.00
808.77	C51H102O5N	e48:1	0.00	0.00	0.00	0.01	0.01	0.00
810.79	C51H104O5N	e48:0	0.00	0.00	0.00	0.03	0.06	0.00
816.71	C51H94O6N	48:4	0.00	0.00	0.00	0.00	0.03	0.01
818.72	C51H96O6N	48:3	0.00	0.00	0.00	0.00	0.00	0.00
820.74	C51H98O6N	48:2	0.00	0.00	0.00	0.06	0.00	0.00
822.75	C51H100O6N	48:1	0.01	0.01	0.01	0.19	0.00	0.01
824.77	C51H102O6N	48:0	0.02	0.03	0.01	0.63	0.14	0.03
830.76	C53H100O5N	e50:4	0.01	0.21	0.00	0.00	0.31	0.33
832.77	C53H102O5N	e50:3	0.00	0.00	0.00	0.00	0.00	0.00
834.79	C53H104O5N	e50:2	0.00	0.00	0.00	0.01	0.01	0.00
836.80	C53H106O5N	e50:1	0.00	0.00	0.00	0.01	0.01	0.00
838.82	C53H108O5N	e50:0	0.00	0.00	0.00	0.02	0.05	0.00
844.74	C53H98O6N	50:4	0.00	0.00	0.00	0.01	0.06	0.01
846.75	C53H100O6N	50:3	0.00	0.02	0.00	0.04	0.03	0.00
848.77	C53H102O6N	50:2	0.03	0.01	0.00	0.11	0.01	0.00
850.78	C53H104O6N	50:1	0.04	0.02	0.00	0.26	0.05	0.02
852.80	C53H106O6N	50:0	0.00	0.02	0.02	0.60	0.03	0.03
856.77	C55H102O5N	e52:5	0.01	0.20	0.01	0.00	0.15	0.35
858.79	C55H104O5N	e52:4	0.00	0.00	0.00	0.00	0.00	0.00
860.80	C55H106O5N	e52:3	0.00	0.00	0.00	0.00	0.06	0.00
862.82	C55H108O5N	e52:2	0.00	0.00	0.00	0.00	0.00	0.00
864.84	C55H110O5N	e52:1	0.00	0.00	0.00	0.00	0.00	0.00
866.85	C55H112O5N	e52:0	0.00	0.00	0.00	0.01	0.02	0.00
868.74	C55H98O6N	52:6	0.00	0.00	0.00	0.00	0.01	0.00
870.75	C55H100O6N	52:5	0.00	0.00	0.00	0.00	0.00	0.00
872.77	C55H102O6N	52:4	0.00	0.00	0.00	0.00	0.00	0.00
874.78	C55H104O6N	52:3	0.00	0.00	0.00	0.01	0.00	0.00
876.80	C55H106O6N	52:2	0.00	0.00	0.00	0.01	0.01	0.00
878.81	C55H108O6N	52:1	0.00	0.00	0.00	0.01	0.01	0.00
880.83	C55H110O6N	52:0/e54:7	0.00	0.00	0.00	0.02	0.16	0.00

882.79	C57H104O5N	e54:6	0.00	0.00	0.00	0.00	0.02	0.01
884.80	C57H106O5N	e54:5	0.00	0.00	0.00	0.00	0.01	0.00
886.82	C57H108O5N	e54:4	0.00	0.00	0.00	0.00	0.02	0.00
888.84	C57H110O5N	e54:3	0.00	0.00	0.00	0.00	0.00	0.00
890.85	C57H112O5N	e54:2	0.00	0.00	0.00	0.00	0.00	0.00
892.87	C57H114O5N	e54:1	0.00	0.00	0.00	0.00	0.00	0.00
894.75	C57H100O6N	54:7/e54:0	0.00	0.00	0.00	0.00	0.00	0.00
896.77	C57H102O6N	54:6	0.00	0.00	0.00	0.00	0.00	0.00
898.78	C57H104O6N	54:5	0.00	0.00	0.00	0.00	0.00	0.00
900.80	C57H106O6N	54:4	0.00	0.00	0.00	0.00	0.00	0.00
902.81	C57H108O6N	54:3	0.00	0.00	0.00	0.00	0.00	0.00
904.83	C57H110O6N	54:2	0.00	0.00	0.00	0.00	0.00	0.00
906.85	C57H112O6N	54:1	0.00	0.00	0.00	0.00	0.00	0.00
908.86	C57H114O6N	54:0/e56:7	0.00	0.00	0.00	0.00	0.00	0.00
910.82	C59H108O5N	e56:6	0.00	0.00	0.00	0.00	0.00	0.00
912.84	C59H110O5N	e56:5	0.00	0.00	0.00	0.00	0.01	0.00
914.85	C59H112O5N	e56:4	0.00	0.00	0.00	0.00	0.00	0.00
916.87	C59H114O5N	e56:3	0.00	0.00	0.00	0.00	0.00	0.01
918.88	C59H116O5N	e56:2	0.00	0.00	0.00	0.00	0.00	0.00
920.90	C59H118O5N	e56:1	0.00	0.00	0.00	0.00	0.00	0.00
922.78	C59H104O6N	56:7/e56:0	0.00	0.00	0.00	0.00	0.01	0.00
924.80	C59H106O6N	56:6	0.00	0.00	0.00	0.00	0.02	0.00
926.81	C59H108O6N	56:5	0.00	0.00	0.00	0.00	0.00	0.00
928.83	C59H110O6N	56:4	0.00	0.00	0.00	0.00	0.01	0.00
930.85	C59H112O6N	56:3	0.00	0.00	0.00	0.00	0.00	0.00
932.86	C59H114O6N	56:2	0.00	0.00	0.00	0.00	0.01	0.00
934.88	C59H116O6N	56:1	0.00	0.00	0.00	0.00	0.00	0.00
936.84	C61H110O5N	e58:7/56:0	0.00	0.00	0.00	0.00	0.02	0.00
938.85	C61H112O5N	e58:6	0.00	0.00	0.00	0.00	0.00	0.00
940.87	C61H114O5N	e58:5	0.00	0.00	0.00	0.00	0.00	0.00
942.88	C61H116O5N	e58:4	0.00	0.00	0.00	0.00	0.00	0.00
944.90	C61H118O5N	e58:3	0.00	0.00	0.00	0.00	0.00	0.00
946.91	C61H120O5N	e58:2	0.00	0.00	0.00	0.00	0.00	0.00
948.93	C61H122O5N	e58:1/58:8	0.00	0.00	0.00	0.00	0.01	0.00
950.81	C61H108O6N	58:7/e58:0	0.00	0.00	0.00	0.00	0.01	0.00
952.83	C61H110O6N	58:6	0.00	0.00	0.00	0.00	0.01	0.00
954.85	C61H112O6N	58:5	0.00	0.00	0.00	0.00	0.01	0.00
956.86	C61H114O6N	58:4	0.00	0.00	0.00	0.00	0.01	0.00
958.88	C61H116O6N	58:3	0.00	0.00	0.00	0.00	0.00	0.00

960.89	C61H118O6N	58:2	0.00	0.00	0.00	0.00	0.00	0.00
962.91	C61H120O6N	58:1	0.00	0.00	0.00	0.00	0.01	0.00
964.87	C63H114O5N	e60:7/58:0	0.00	0.00	0.00	0.00	0.00	0.00
966.88	C63H116O5N	e60:6	0.00	0.00	0.00	0.01	0.00	0.00
968.90	C63H118O5N	e60:5	0.00	0.00	0.00	0.00	0.00	0.00
970.91	C63H120O5N	e60:4	0.00	0.00	0.00	0.00	0.01	0.00
972.93	C63H122O5N	e60:3	0.00	0.00	0.00	0.01	0.01	0.00
974.94	C63H124O5N	e60:2	0.00	0.00	0.00	0.00	0.00	0.00
976.83	C63H110O6N	60:8/e60:1	0.00	0.00	0.00	0.00	0.01	0.00
978.85	C63H112O6N	60:7/e60:0	0.00	0.00	0.00	0.00	0.00	0.00
980.86	C63H114O6N	60:6	0.00	0.00	0.00	0.00	0.01	0.00
982.88	C63H116O6N	60:5	0.00	0.00	0.00	0.00	0.00	0.00
984.89	C63H118O6N	60:4	0.00	0.00	0.00	0.00	0.00	0.00
986.91	C63H120O6N	60:3	0.00	0.00	0.00	0.00	0.00	0.00
988.92	C63H122O6N	60:2	0.00	0.00	0.00	0.00	0.01	0.00
990.94	C63H124O6N	60:1	0.00	0.00	0.00	0.00	0.00	0.00
Total NL 245 14:0 acyl containing		Total NL 245 14:0 acyl containing	0.21	0.69	0.14	2.88	2.53	1.01
	Cholesterol		20.12	30.25	40.61	132.20		
	Acylcarnitine (AC) (pmol)							
370.3	14:1		0.0004	0.0005	0.0006	0.0009		
372.31	14:0		0.0010	0.0009	0.0012	0.0014		
398.33	16:1		0.0008	0.0010	0.0012	0.0014		
400.34	16:0		0.0012	0.0015	0.0022	0.0050		
414.32	16:1-OH		0.0009	0.0001	0.0013	0.0022		
416.34	16:0-OH		0.0001	0.0001	0.0002	0.0003		
424.34	18:2		0.0009	0.0005	0.0017	0.0020		
426.36	18:1		0.0019	0.0025	0.0029	0.0048		
428.37	18:0		0.0014	0.0055	0.0029	0.0136		
442.35	18:1-OH		0.0004	0.0002	0.0008	0.0021		
444.37	18:0-OH		0.0005	0.0001	0.0009	0.0029		
	Total Acylcarnitine		0.0095	0.0131	0.0234	0.0451		
	Ceramide (CER)							
536.5	N16:0		0.1456	0.8123	0.224	1.072		
562.52	N18:1		0.0587	0.7485	0.112	0.917		
564.54	N18:0		0.0452	0.6874	0.060	0.899		
646.61	N24:1		0.0587	0.0052	0.079	0.015		
648.63	N24:0		0.0354	0.0235	0.066	0.058		

	Total Ceramide	0.3436	2.2769	0.542	2.961

Supplementary Table 4. Detail description of 60% high fat diet and regular chow diet.

**Class description**	**Ingredient**	**Grams**
**Protein**	Casein, Lactic, 30Mesh	200
**Protein**	Cystine, L	3
**Carbohydrate**	Lodex 10,	125
**Carbohydrate**	Fine granulated Sucrose	72.8
**Fiber**	Solka Floc, FCC200	50
**Fat**	Lard	245
**Fat**	Soybean oil, USP	25
**Mineral**	S10026B	50
**Vitamin**	Choline bitartrate	2
**Vitamin**	V10001C	1
**Dye**	Dye Blue FD&C #1, Alum. Lake 35-42%	0.05

**Caloric information**	
**Protein**	20%
**Fat**	60%
**Carbohydrate**	20%
**Energy density**	5.21%

Regular chow diet composition.

Nutrients	%100g
Carbohydrate	48.8
Protein	21.0
Fat	3.0
Calcium	0.8
Phosphorous	0.4
Fiber	5.0
Moisture	13.0
Ash	8.0
Total energy (kcal/100g)	306.2

Supplementary Table 5. List of mouse primers used in the study

	Target name	Forward (Primer sequence 5' – 3')	Reverse (Primer sequence 5' – 3')
1.	mAhR	GGCTTTCAGCAGTCTGATGTC	CATGAAAGAAGCGTTCTCTGG
2.	mIRS2	GTCCAGGCACTGGAGCTTT	GCTGGTAGCGCTTCACTCTT
3.	mIRS1	TTTGAAGACCATAACCCACCAC	ATTACACCAGTTCGTCCCTTTC
4.	mIRS2	TCCAGAACGGCCTCAACTAT	AGTGATGGGACAGGAAGTCG
5.	mLDLR	TCAGACGAACAAGGCTGTCC	CCATCTAGGCAATCTCGGTCTC
6.	mPtpn1	GGAACTGGGCGGCTATTTACC	CAAAAGGGCTGACATCTCGGT
7.	mJUN	TTCCTCCAGTCCGAGAGCG	TGAGAAGGTCCGAGTTCTTGG
8.	mCyp1a1	GCCACATCCGGGACATCAC	GCTGGACATTGGCATTCTCGT
9.	mCyp1a2	AGTACATCTCCTTAGCCCCAG	GGTCCGGGTGGATTCTTCAG
10.	mCyp1b1	CCACCAGCCTTAGTGCAGAC	GGCCAGGACGGAGAAGAGT
11.	mTNFα	CACAGAAAGCATGATCCGCGACGT	CGGCAGAGAGGAGGTTGACTTTCT
12.	mIL6	GAGAGGAGACTTCACAGAGGATAC	GTACTCCAGAAGACCAGAGG
13.	mIL10	TTTGAATTCCCTGGGTGAGAA	GGAGAAATCGATGACAGCGC
14.	mLep	GTGGCTTTGGTCCTATCTGTC	CGTGTGTGAAATGTCATTGATCC
15.	mPparg	TCGCTGATGCACTGCCTATG	GAGAGGTCCACAGAGCTGATT
16.	mSrebf1	TGACCCGGCTATTCCGTGA	CTGGGCTGAGCAATACAGTTC
17.	mTg	TGTGGGTCTCTACTTTGTTGAGC	CGGAGTGGCTGTGCATCTAC
18.	mB2m	TTCTGGTGCTTGTCTCACTGA	CAGTATGTTCGGCTTCCCATTC
19.	mINS1	CACTTCCTACCCCTGCTGG	ACCACAAAGATGCTGTTTGACA
20.	miR-375	TTTGTTCGTTCGGCTCGCGTGA	Qiagen Universal primer
21.	16S	AGGAACTCCGATTGCGAAGG	TCGGTTTACTGCGTGGACTACC
22.	Ppia	GAGCTGTTTGCAGACAAAGTTC	CCCTGGCACATGAATCCTGG

Supplementary Table 6. List of antibodies used in the study

S. No.	Target	Source	Cat. No.	Application
1.	AHR	Santacruz	sc133088	Western blot
2.	pAHR	ThermoScientific	PA5-38404	Western blot/IF
3.	pIRS-2	Abcam	Ab3690	Western blot
4.	CD63	Biolegend	143902	Western blot/Flow cytometry/IF
5.	CD63	Novus Biologicals	NBP2-32830	Pull down
6.	A33	Biorybt	orb15687	Western blot/Flow cytometry
7.	PEMT	ThermoScientific	PA5-42383	Western blot
8.	Albumin	Cell Signaling	4929S	Western blot/IF
9.	F4/80	ThermoScientific	11-4801-82	Flow cytometry
10.	F4/80	Abcam	Ab240946	IF
11.	TNF-α	ThermoScientific	39-8321-60	ELISA
12.	TNF-α	R&D Systems	AF-410-SP	Neutralization
13.	IL-6	R&D Systems	MAB406	ELISA
14.	IL-6	R&D Systems	MAB406	Neutralization
15.	CD14	eBioscience	12-0141-81	Flow cytometry
16.	GAPDH	Santacruz	sc47724	Western blot
17.	Histone	Santacruz	sc10807	Western blot
18.	β-Actin	Santacruz	sc47778	Western blot
19.	IgG	Santacruz	sc65662	Pull down
20.	MHCII	Abcam	Ab180779	Western blot
21.	CD9	Abcam	Ab92726	Western blot
22.	CD81	Abcam	Ab109201	Western blot
23.	CD31	Abcam	Ab24590	IF
24.	LVYE-1	ThermoScientific	14-0443-82	IF
25.	PI3K	Epitomics	3295-1	Western blot
26.	AKT	NEB	9272	Western blot
27.	pAKT	Epitomics	2118-1	Western blot

Supplementary Table 7. Kits and Array used in the study

Mouse miRNA array	Qiagen	331223
Cytokine array	R&D systems	AY028
Insulin Signaling array	Qiagen	PAMM-030ZE

Supplementary Table 8. Summary of the statistics for the figures with statistical significance

Figure	Statistical method	Statistical significance between groups
Fig. 1b	Two-tailed *t* test	L-Exo vs H-Exo
Fig. 1d	Two-tailed *t* test	L-Exo vs H-Exo
Fig. 1f	Two-tailed *t* test	Healthy-Exo vs T2D-Exo
Fig. 2a, b & c	One-way ANOVA with a Bonferroni post hoc test	* PBS vs H-Exo
Figure 2d-i	Two-way ANOVA with a Tukey post hoc test	As indicated in figure panels
Fig. 2j	One-way ANOVA with a Bonferroni post hoc test	* PBS vs T2D-Exo
Fig. 2k; left panel	One-way ANOVA with a Bonferroni post hoc test	* PBS vs H-Exo
Fig. 2k; right panel	One-way ANOVA with a Bonferroni post hoc test	* PBS vs T2D-Exo
Fig. 2l	One-way ANOVA with a Bonferroni post hoc test	* PBS vs H-Exo 12M Ab^-
Fig. 2m	One-way ANOVA with a Bonferroni post hoc test	* PBS vs L-Exo$^{\text{lipids PC+}}$
Fig. 3b & c	Two-tailed *t* test	L-Exo vs H-Exo
Fig. 5k GTT	One-way ANOVA with a Bonferroni post hoc test	* AhRKO H-Exo vs WT H-Exo
Fig. 5k ITT	One-way ANOVA with a Bonferroni post hoc test	* AhRKO H-Exo vs WT H-Exo
Fig. 5n	Student t test (Two-tailed)	* AhRKO Ad-GFP-H-Exo vs AhRKO Ad-AhR-H-Exo

Supplemental Reference

[1]1 Coliva, G. *et al.* Sphingomyelins Prevent Propagation of Lipid Peroxidation-LC-MS/MS Evaluation of Inhibition Mechanisms. *Molecules* **25**, doi:10.3390/molecules25081925 (2020).
