# Supplementary material for: High-fat diet-induced upregulation of exosomal phosphatidylcholine contributes to insulin resistance
Source: Nat Commun. 2021 Jan 11;12:213. doi: 10.1038/s41467-020-20500-w (PMC7801461; doi:10.1038/s41467-020-20500-w)
Supplement: Supplementary file 3 — Description of Additional Supplementary Files [file 41467_2020_20500_MOESM3_ESM.pdf]

Description of Additional Supplementary Files

File Name: Supplementary Data 1
Description: Lipids detected by triple quadrupole MS in plasma exosomes (PL Exo) and FACS sorted A33+ IEC cells from RCD and HFD mice (12 months of their respective diets)

File Name: Supplementary Data 2
Description: Plasma insulin concentration (ng/ml) during GTT.

File Name: Supplementary Data 3
Description: Lipids detected by triple quadrupole MS in TLC extracted remainder lipids after PC band depletion and PC band.

File Name: Supplementary Data 4
Description: Cytokine array performed for plasma and white adipose tissue extracts derived from mice treated with L-Exo or H-Exo.

File Name: Supplementary Data 5
Description: The lipids identified by lipidomics analysis with all of the relevant information.
