# Supplementary material for: High-fat diet-induced upregulation of exosomal phosphatidylcholine contributes to insulin resistance
Source: Nat Commun. 2021 Jan 11;12:213. doi: 10.1038/s41467-020-20500-w (PMC7801461; doi:10.1038/s41467-020-20500-w)
Supplement: Supplementary file 9 — Reporting Summary [file 41467_2020_20500_MOESM9_ESM.pdf]

nature research

Corresponding author(s): Dr. Huang-Ge Zhang

Last updated by author(s): Nov 20, 2020

Reporting Summary

Nature Research wishes to improve the reproducibility of the work that we publish. This form provides structure for consistency and transparency in reporting. For further information on Nature Research policies, see our Editorial Policies and the Editorial Policy Checklist.

Statistics

For all statistical analyses, confirm that the following items are present in the figure legend, table legend, main text, or Methods section.

n/a	Confirmed	
☐	☒	The exact sample size (*n*) for each experimental group/condition, given as a discrete number and unit of measurement
☐	☒	A statement on whether measurements were taken from distinct samples or whether the same sample was measured repeatedly
☐	☒	The statistical test(s) used AND whether they are one- or two-sided<br>*Only common tests should be described solely by name; describe more complex techniques in the Methods section.*
☒	☐	A description of all covariates tested
☐	☒	A description of any assumptions or corrections, such as tests of normality and adjustment for multiple comparisons
☐	☒	A full description of the statistical parameters including central tendency (e.g. means) or other basic estimates (e.g. regression coefficient) AND variation (e.g. standard deviation) or associated estimates of uncertainty (e.g. confidence intervals)
☐	☒	For null hypothesis testing, the test statistic (e.g. *F*, *t*, *r*) with confidence intervals, effect sizes, degrees of freedom and *P* value noted<br>*Give P values as exact values whenever suitable.*
☒	☐	For Bayesian analysis, information on the choice of priors and Markov chain Monte Carlo settings
☒	☐	For hierarchical and complex designs, identification of the appropriate level for tests and full reporting of outcomes
☒	☐	Estimates of effect sizes (e.g. Cohen's *d*, Pearson's *r*), indicating how they were calculated

Our web collection on statistics for biologists contains articles on many of the points above.

Software and code

Policy information about availability of computer code

Data collection	All raw data were included as supplementary or extended tables or provided in source data file.
Data analysis	Graph Pad Prism 7.0 were used to calculate the statistical significance among the groups. FACS DIVA software v8.0.2 was used to acquire the data and FlowJo software v7.6 (Tree Star Inc., Ashland, OR) was to analyze the data. Proteome Discoverer v1.4.1.114 (Thermo) was used to analyze the data collected by the mass spectrometer. HLImage++ was used to analyze cytokine array. TraceDrawer was used to acquire and analyzed the SPR results.

For manuscripts utilizing custom algorithms or software that are central to the research but not yet described in published literature, software must be made available to editors and reviewers. We strongly encourage code deposition in a community repository (e.g. GitHub). See the Nature Research guidelines for submitting code & software for further information.

Data

Policy information about availability of data

All manuscripts must include a data availability statement. This statement should provide the following information, where applicable:

- Accession codes, unique identifiers, or web links for publicly available datasets
- A list of figures that have associated raw data
- A description of any restrictions on data availability

All required figures were included in the manuscript. Some data figures were also included as supplementary or extended figures. The findings of this study have been deposited in the NCBI GEO database under accession number GSE156848 and publicly available. MS proteomics data have been deposited to the ProteomeXchange Consortium via the PRIDE partner repository with the dataset identifier PXD022641.

Field-specific reporting

Please select the one below that is the best fit for your research. If you are not sure, read the appropriate sections before making your selection.

- [x] Life sciences
- [ ] Behavioural & social sciences
- [ ] Ecological, evolutionary & environmental sciences

For a reference copy of the document with all sections, see nature.com/documents/nr-reporting-summary-flat.pdf

Life sciences study design

All studies must disclose on these points even when the disclosure is negative.

Sample size	Sample sizes for animal studies were chosen using one-way ANOVA upto 3 groups as described in Teng et al., 2018. Experiments other than animals involved, were also chosen using the method described by Teng et al., 2017 & 2018.
Data exclusions	No data were excluded from the analysis.
Replication	All experiments were independently repeated at least three times and were successful. Control and experimental groups were tested under identical conditions.
Randomization	Animals or individuals were randomly assigned but matched with their ages and sex. Cell culture samples were assigned randomly, with control and experimental groups analyzed in identical conditions to minimize potential covariates.
Blinding	The researchers were blinded during the data collection and analysis.

Reporting for specific materials, systems and methods

We require information from authors about some types of materials, experimental systems and methods used in many studies. Here, indicate whether each material, system or method listed is relevant to your study. If you are not sure if a list item applies to your research, read the appropriate section before selecting a response.

Materials & experimental systems

n/a	Involved in the study	
☐	☒	Antibodies
☐	☒	Eukaryotic cell lines
☒	☐	Palaeontology and archaeology
☐	☒	Animals and other organisms
☐	☒	Human research participants
☒	☐	Clinical data
☒	☐	Dual use research of concern

Methods

n/a	Involved in the study	
☒	☐	ChIP-seq
☐	☒	Flow cytometry
☒	☐	MRI-based neuroimaging

Antibodies

Antibodies used	Supplementary table for antibodies and their resources was added into supplementary information. Antibody dilution factor mentioned in the method section.
Validation	Antibody AhR (Bacsi et al., 1996), Albumin (Cai Zhang et al., 2018) CD63 (Miller et a., 2019 for pull down), PEMT (Kim et al., 2018), GAPDH (De Pace et al., 2020), Histone (Huo et al., 2010), Beta actin (Zou et al., 2021), MHCII (Biglari et al., 2019), CD9 (Wolf et al., 2020), CD81 (Chen et al., 2020), CD31 (Fan et al., 2020), LVYE-1 (Olga et al., 2019), PI3K, AKT (Jinchuan et al., 2020), TNF-alpha and IL-6 (Li et al., 2020), pAhR, pIRS2, A33 and pAKT were used in the western blot and immunofluoresecence. The validation of these antibodies mentioned on the source website as reference quoted.

Eukaryotic cell lines

Policy information about cell lines

Cell line source(s)	All cell lines (FL83B, C2C12, 3T3-L1 and HepG2) used in the study, were purchased from American Type Culture Collection (ATCC).
Authentication	3T3-L1 were differentiated with insulin as per the established protocol by Shona et al., 2015. C2C12 were differentiated with insulin as per the established protocol by Katherine et al., 2011. FL83B and HepG2 authenticated cell lines obtained from ATCC.
Mycoplasma contamination	Cell lines were checked for Mycoplasma contamination. No cell lines were used with Mycoplasma contamination.

Commonly misidentified lines (See ICLAC register)	No misidentified lines used in the study.

Animals and other organisms

Policy information about studies involving animals; ARRIVE guidelines recommended for reporting animal research

Laboratory animals	8 - 12 weeks old male C57BL/6, Germ free C57BL/6 and AhR Knock out C57BL/6 mice were used in the study. All animals were housed in a pathogen-free animal facility at the University of Louisville. All animals were housed at ambient temperature of 18-23 degree C and 40-60% humidity with food access ad libitum.
Wild animals	The study did not involve any wild animals.
Field-collected samples	The study did not involve samples collected from the field.
Ethics oversight	Animal care was performed following the Institute for Laboratory Animal Research (ILAR) guidelines, and all animal experiments were conducted in accordance with protocols approved by the University of Louisville Institutional Animal Care and Use Committe (Louisville KY).

Note that full information on the approval of the study protocol must also be provided in the manuscript.

Human research participants

Policy information about studies involving human research participants

Population characteristics	25 to 45 years old (all males) healthy subjects and Type 2 diabetes (T2D) patients were included in the study. No healthy volunteers had a history of chronic gastrointestinal disease. All volunteers were recruited from the University of Louisville Hospital, Louisville, Kentucky, USA. Type 2 diabetes was diagnosed according to the American Diabetes Association diagnostic criteria (American Diabetes Association 2012). All clinical fecal samples were collected from patients in the outpatient endocrinology clinic at University of Louisville Hospital.
Recruitment	No self selection bias or any other bias was adopted during the recruitment of individuals in the study. All volunteers were recruited from the University of Louisville Hospital, Louisville, Kentucky, USA. Type 2 diabetes was diagnosed according to the American Diabetes Association diagnostic criteria (American Diabetes Association 2012).
Ethics oversight	Approval for the study was granted by the University of Louisville Research Ethics Committee.

Note that full information on the approval of the study protocol must also be provided in the manuscript.

Flow Cytometry

Plots

Confirm that:

- [x] The axis labels state the marker and fluorochrome used (e.g. CD4-FITC).
- [x] The axis scales are clearly visible. Include numbers along axes only for bottom left plot of group (a 'group' is an analysis of identical markers).
- [x] All plots are contour plots with outliers or pseudocolor plots.
- [x] A numerical value for number of cells or percentage (with statistics) is provided.

Methodology

Sample preparation	Samples were prepared according to published articles from our laboratory.
Instrument	BD FACSCanto flow cytometer (BD Biosciences, San Jose, CA). Ref. No. 337175
Software	FACS DIVA software v8.0.2 was used to acquire the data and FlowJo software v7.6 (Tree Star Inc., Ashland, OR) was to analyze the data.
Cell population abundance	Cell populations were identified by their specific markers. At least 50000 cells were acquired for the analysis.
Gating strategy	Unstained, antibody controls and FMO were used for the gating strategy.

- [x] Tick this box to confirm that a figure exemplifying the gating strategy is provided in the Supplementary Information.
